# Symposium Mammographicum Conference 2021: Virtual. 7-10 February 2021

**DOI:** 10.1186/s13058-021-01443-6

**Published:** 2021-07-06

## O1.1 Chasing calcification -- are we performing too many diagnostic stereotactic biopsies for the assessment of microcalcifications? An audit of practice at the Nottingham Breast Institute

### Ketan Jethwa**,** Lisa Hamilton

#### Nottingham Breast Institute, Nottingham, United Kingdom

**Introduction**: Stereotactic breast biopsies are performed for the assessment of indeterminate mammographic abnormalities where no target can be identified on ultrasound scanning.

**Methods:** A retrospective audit of stereotactic biopsies performed at the Nottingham Breast Institute was undertaken to quantify the number of malignant and benign biopsy results. Patient demographics, characteristics of the mammographic abnormality and pathological diagnosis were extracted. This analysis focussed on patients recalled from the National Health Service Breast Screening Programme (NHSBSP).

**Results:** Between November 2018 and October 2019 68 stereotactic breast biopsies were performed. The majority were for patients recalled from the NHS BSP (52, 76.5%). Mean age of patients in this cohort was 58.8 years. 33 (63.5%) malignant biopsies and 19 (36.5%) benign biopsies were obtained. Within the NHSBSP recall group 40 (76.9%) patients were recalled for indeterminate microcalcifications (M3/M4). Within this group 26 (65%) malignant and 14 (35%) benign biopsy results were obtained. Mean age of patients in the malignant and benign biopsy group were 57.2 and 61.1 years respectively. Mean size of microcalcifications in the malignant and benign biopsy group were 30.4mm and 18.9mm respectively. Histograms demonstrate overlap between groups with the majority of microcalcifications measured between 10mm and 20mm. Malignant calcifications tend to be spread over a greater distance and there is wider frequency distribution in this group.

**Conclusion:** Two thirds of stereotactic biopsies performed for NHSBSP recall patients for indeterminate calcifications yield a malignant diagnosis in our practice.

## O1.2 B3 lesions -- VAE to B5b or not to B5b, a DGH experience

### Julia Yemm, Amanjot Karuppiah, Constantine Fragkoulakis

#### **Correspondence:** Julia Yemm

##### Sherwood Forest Hospitals NHS Foundation Trust, Nottingham, United Kingdom

**Background / Objectives:** The aim of this audit was to evaluate the local implementation of the B3 breast lesion pathway using NHSBSP Guidance 2016 as the audit standard by assessing the following points.

1. Does the local B3 breast biopsy rate meet the standard?

2. Is the correct procedure performed for each B3 biopsy case?

3.What is the rate of upgrade?

4. Do these patients have the correct follow up as per the standard?

**Methods:** Using the CRIS system, the total number of breast biopsies, both screening and symptomatic, performed at KMH between January 2018 and July 2019 was obtained. The histopathology department provided the results data for these biopsies. Further procedures and final pathology was collected from CRIS and ICE systems. Meridian was used to collect and analyse the data.

**Results:** The total number of biopsies during this period was 1028.

71 patients (6.9%) had a B3 result, meeting the standard (3.3%- 12.6%).

50% were screening and 50% were symptomatic patients.

71% were Ultrasound guided biopsies and 29% were stereotactic 10g vacuum biopsies.

100% of the patients had a further procedure as recommended by the MDT- 50% had a Vacuum excision and 50% had diagnostic surgical excision.

The upgrade rate of 24% meets the standard. (9.9%-35.1%). 13.6% DCIS and 10.6% B5b malignant.

**Conclusions:** The standard is met for B3 biopsy rate, recommended management, upgrade and follow up. There is a higher number of diagnostic surgical excisions than expected, but the standards only apply to screening patients.

## O1.3 Risk of breast cancer following detection of breast lesions of uncertain malignant potential: A 23 year retrospective review

### Nerys Forester, Christopher Ng, Nadia McAllister

#### **Correspondence:** Nerys Forester

##### Newcastle Hospitals, Newcastle, United Kingdom

**Purpose:** B3 breast lesions are associated with an increased risk of subsequent breast malignancy. Current management includes 5 year enhanced mammographic follow up post diagnosis. This study compares incidence of malignancy following B3 diagnosis, to that in a group of benign lesions

**Methods:** Retrospective, single centre, review of subsequent breast cancers in screen detected B3 lesions between 1995-2008, compared with screen detected benign lesions identified between 1995-6. Follow up to December 2018

**Results:** Between 1995-2008, 188 B3 lesions identified (41% RS/CSL 29% ADH 39% papilloma 8% LN 1% other). Control group comprised 161 benign lesions, recalled from screening with benign biopsy (B2) or cytology (C2) assessment outcomes. All subjects were followed up for 10-23 years. Subsequent breast cancer occurred in 21 (11.2%) of the B3 group and 12 (7.5%) of controls. Breast cancer occurred between 1 and 17 years following B3 diagnosis, often not at the same site as the initial B3 lesion. Kaplan Meier curves showed no significant difference in rate of breast cancer between the groups

**Conclusion:** Risk of subsequent breast cancer following B3 lesion diagnosis is not significantly increased compared to a group of screened women with benign lesions, and is equivalent to the risk of cancer expected in a screened population (8-9%). Cancers occurred at a wide range of time points following B3 diagnosis, and not predominately within the first 5 years. As such, enhanced mammographic surveillance for 5 years within this group of women may not be justified, and only serve to increase patient anxiety.

## O1.4 The prognostic impact of mode of detection of axillary metastases for women with invasive breast cancer

### Kirsty McNeil, Andy Evans, Jane Macaskill, Colin Purdie

#### NHS Tayside, Dundee, United Kingdom

**Aim:** To identify the breast cancer specific survival (BCSS) associated with nodal metastasis identified by axillary core biopsy (ACB), and by sentinel node biopsy (SNB) compared with patients with no nodal involvement. A further aim is to identify the prognostic effects of different axillary ultrasound features and amount of tumour in ACB specimens.

**Methods:** Consecutive invasive cancers were identified from a prospective database of all ultrasound (US) lesions undergoing breast biopsy over 5 yrs. The three study groups were those with nodal metastasis identified by ACB, and those undergoing immediate surgery with positive and negative SNB. The BCSS of patients with a positive ACB was compared those with positive and negative SNB’s using Kaplan Meier survival curves. The prognostic effect of axillary US and ACB findings were similarly assessed.

**Results:** 967 patients were included with mean follow-up of 6 yrs. 90 breast cancer deaths occurred. Breast cancer death occurred in 4% of those with a negative SNB, 11% with a positive SNB and 26% of those with a positive ACB. BCSS was significantly different between the groups (p<0.001) with hazard ratio compared with the negative SLNB group for women with a positive SLNB of 2.5 (95% CI 1.3-4.6) and 7.8 (95% CI 4.4-13.7) for those with a positive ACB. Axillary US findings and assessment of the amount of tumour in the ACB did not influence survival.

**Conclusion:** Women with a positive ACB have a markedly worse BCSS compared to those with a positive SNB.

## O1.5 Contrast enhanced digital breast tomosynthesis for the monitoring of response to neoadjuvant chemotherapy: Preliminary results

### Sarah Savaridas^1^, Violet Warwick^1^, Sarah Vinnicombe^2^, Colin Purdie^1^, Andy Evans^1^

#### **Correspondence:** Sarah Savaridas

##### ^1^University of Dundee, Dundee, United Kingdom; ^2^NHS Gloucester Foundation Trust, Gloucester, United Kingdom

**Background:** Monitoring response to neoadjuvant chemotherapy (NACT) is essential. MRI, the gold-standard technique, is expensive, time-consuming, difficult to access and can be poorly tolerated. Contrast-enhanced breast tomosynthesis (CE-DBT) combines functional information on vascularity from contrast enhanced mammography (CESM) with structural information obtained at tomosynthesis. We seek to compare CE-DBT with MRI for accuracy of response assessment and patient experience.

**Methods:** This is a prospective imaging-comparison pilot study of adult female patients with breast cancer undergoing NACT. Participants undergo CE-DBT alongside MRI, before, during and after completion of NACT. Participant questionnaires are completed after initial and end-of-treatment imaging. Comparison between end-of-treatment imaging and pathology and between CE-DBT and MRI will be made.

**Results:** 17 women (18 cancers) are recruited, target 25 women. Ten have completed treatment with surgical pathology available, one withdrew, thus CE-DBT-pathological correlation is available for 10 tumours (9 women). 47 questionnaires were completed (26 post-CE-DBT, 21 post-MRI).

CESM accurately predicted pathological complete response in 5 cases, with one false negative (6mm residual grade 1 IDC). Of the remaining four tumours, CESM was accurate to within 2mm in two cases; of the two residual multicentric tumours CESM over-estimated one case and under-estimated another.

On 19/24 (79%) of occasions women preferred CE-DBT to MRI. Overall experience was significantly better for CE-DBT (*p*<0.005); rated as excellent, good and fair in 17,9,0 cases opposed to 8,7,6 for MRI.

**Conclusions:** Early indications suggest CE-DBT may be a reliable monitoring technique, preferred by patients. Full analysis and comparison with MRI results will be performed.

## O2.1 Estimating the cost impact of including Magnetic Resonance Imaging (MRI) in the National Health Service Breast Screening Programme (NHSBSP) for population-risk women in England

### Hesam Ghiasvand^1^, Claire Hulme^1^, Sam Harding^2^, Sadie McKeown-Keegan^2^, Rebecca Geach^2^, Lyn Jones^2^

#### **Correspondence:** Claire Hulme

##### ^1^University of Exeter, Exeter, United Kingdom; ^2^North Bristol NHS Trust, Bristol, United Kingdom

**Background:** Early diagnosis of breast cancer saves lives ^[1,2]^ and is the aim of the NHSBSP ^[3]^. However, mammographic screening programmes result in disputed levels of both over-diagnosis and under-diagnosis ^[4,5]^. Breast MRI is better at finding cancers but scanning takes longer and needs senior medical staff to interpret it. The aim of this paper is to estimate the budget impact to the NHS of the use of MRI in breast cancer population screening. The objectives are: To estimate the cost per extra detected breast cancer of (a) using MRI alongside mammography vs. mammogram alone, and (b) MRI alone vs. mammography alone.

**Methods:** A decision analytical model taking the perspective of the NHS in England using a three-year time horizon and price year 2019 was developed. Model parameters were taken from literature.

**Results:** The analysis showed increased costs associated with the use of MRI both for the inclusion of MRI with mammography vs. mammography alone and for MRI alone vs. mammography. The cost per extra detected cancer was between £22,170-£14,642 and £17,144-£9,859 respectively.

**Conclusions:** To reduce breast cancer mortality, we need a cost-effective screening test that preferentially finds aggressive cancers that are not well seen on mammogram and works well for all women. MRI is effective but the cost may be prohibitive. Further research, to determine the cost effectiveness of alternative versions of breast MRI, such as FAST MRI, which reduce scanning times and can be interpreted by the same professionals who interpret mammograms, is needed.

**Funding:** NIHR RfPB (ISRCTN 16624917)

[1]. Saadatmand S, Bretveld R, Siesling S, Tilanus-Linthorst MMA. Influence of tumour stage at breast cancer detection on survival in modern times: Population based study in 173 797 patients. BMJ. 2015;351. [2]. Massat NJ, Sasieni PD, Tataru D, Parmar D, Cuzick J, Duffy SW. Explaining the Better Prognosis of Screening- Exposed Breast Cancers : Influence of Tumor Characteristics and Treatment. Cancer Epidemiol Biomarkers Prev. 2015;25(3):479–88. [3]. NHS Digital. Breast Screening Programme, England [Internet]. NHS Digital. 2018. Available from: https://digital.nhs.uk/data-and-information/publications/statistical/breast-screening-programme/breast-screening-programme-england---2016-17#[4]. Autier P, Boniol M, Koechlin A, Pizot C, Boniol M. Effectiveness of and overdiagnosis from mammography screening in the Netherlands: population based study. BMJ. 2017;359:j5224. [5]. Marmot M. Review The benefits and harms of breast cancer screening: an independent review Independent UK Panel on Breast Cancer Screening*. Lancet [Internet]. 2012;380:1778–86. Available from: http://dx.doi.org/10.1016/ (accessed March 2020)

## O2.2 Expression of the gadolinium transporters SLCO1B1 and SLCO1B3 in breast cancers: a potential role in influencing tumour enhancement on MRI

### Rachel Sutherland^1^, Annette Meeson^1^, Simon Lowes^2^

#### **Correspondence:** Rachel Sutherland

##### ^1^Newcastle University, Newcastle, United Kingdom; ^2^Queen Elizabeth Hospital, Gateshead Health NHS Foundation Trust, Gateshead, United Kingdom

**Purpose:** To investigate the expression of the gadolinium-transporting solute carriers SLCO1B1 and SLCO1B3 in breast cancer. Both transporters were previously thought to be liver-specific and are known to influence the enhancement characteristics of liver lesions on MRI. If these transporters are also expressed in breast cancer cells it may influence the enhancement characteristics of breast cancers on MRI.

**Methods:** Transporter expression was investigated in two breast cancer cell lines, MCF-7 (hormone responsive) and MDA-MB-231 (hormone non-responsive), and in tumour samples from four patients (all ER positive invasive ductal carcinoma). Semi-quantitative and real-time PCR (sq-PCR and RT-PCR) was used in both cell lines followed by bulk-cell RNA/single-cell RNA sequencing. Protein expression was investigated by immunocytochemistry. In the tumour samples, expression was investigated by sq-PCR and RT-PCR.

**Results:** SLCO1B1 and SLCO1B3 showed differential expression between the MCF-7 and MDA-MB-231 cell lines, with considerably higher expression in MDA-MB-231 cells than MCF-7 cells both at the gene and protein levels. Expression of SLCO1B1 was detected in all four patient tumours at variable levels. SLCO1B3 was detectable in 2 of 4 patient tumours, and when present it was detected at high levels.

**Conclusions:** Confirming the presence and differential expression of SLCO1B1 and SLCO1B3 in breast cancer cells suggests that these gadolinium transporters may play a role in tumour enhancement on MRI. Further characterisation of expression levels in different tumour types, such as invasive lobular carcinomas, may help to substantiate this and provide a basis to develop studies to investigate its potential relevance in clinical practice.

## O2.3 Determination of onset fatigue while reading Digital Breast Tomosynthesis (DBT) cases

### Mitchell Searjeant^1^, Peter Phillips^2^, Alastair Gale^3^, Dorina Roy^1^, Amanda Koh^1^, Ellhia Sudin^1^, Nisha Sharma^4^, William Teh^5^, Humaira Khan^6^, Michael Michell^7^

#### **Correspondence:** Mitchell Searjeant

##### ^1^The University of Nottingham, Nottingham, United Kingdom; ^2^Cumbria University, Carlisle, United Kingdom; ^3^Loughborough University, Loughborough, United Kingdom; ^4^The University of Leeds, Leeds, United Kingdom; ^5^North London Breast Screening, London, United Kingdom; ^6^Sandwell and West Birmingham NHS Trust, West Bromwich, United Kingdom; ^7^King's College London, London, United Kingdom

**Objectives:** To identify the onset of fatigue during DBT reading through the use of eye tracking and analysing blinking behaviour. The multiple image slices associated with DBT makes reading a longer and potentially more strenuous process when compared with FFDM. The aim is to provide an informed recommendation regarding the appropriate amount of time to spend reading DBT cases to reduce fatigue onset.

**Methods:** Participants read a set of 40 DBT cases in one session while their visual search behaviour was recorded using a non-intrusive eye tracker. Our focus was on analysing the ‘Eyelid Opening’ signal generated by the eye tracker and detecting blinks through the development of a dedicated software. This allowed us to identify certain aspects of blinking behaviour, namely blink duration. Blinks were classified by their duration into ultra-short, short and long blinks and micro-sleeps and then correlated with different states of vigilance and fatigue.

**Results:** A statistical statistically significant difference in the average of blink durations for the 20th cases (Mean: 143 ms) and the 40th cases (Mean: 228 ms,) p = 0.0013) across participants was found. Moreover, analyses Analysis of the eyelid opening aperture across participants showed that readers’ tend towards a smaller eyelid aperture gets progressively smaller (< 10 mm) in the third hour of reading compared with larger eyelid apertures (>12 mm) in the first two hours of reading.

**Conclusions:** Increased blink durations and reduced eyelid apertures were found after 20 cases were read, indicating that individuals begin to exhibit signs of visual fatigue.

## O3.1 Smoking is an independent negative predictor of mammography attendance in women eligible for breast screening

### Naoise C Synnott^1^, Patricia Fitzpatrick^2^

#### **Correspondence:** Patricia Fitzpatrick

##### ^1^Division of Cancer Epidemiology and Genetic, National Cancer Institute, Rockville, Maryland, USA; ^2^University College Dublin, Dublin, Ireland

**Background:** BreastCheck is the national breast screening programme in Ireland; in 2014-2015 the eligible age group was 50-64. The aim of this study was to identify behavioural lifestyle predictors of mammography attendance in Irish women aged 50 - 64.

**Methods:** Data from the Irish Longitudinal Study on Ageing (TILDA) Wave 3, conducted in 2014-2015, was used. Multivariable logistic regression was employed to identify independent predictors of mammography attendance.

**Results:** There were 3,575 female participants in TILDA Wave 3; 1,750 were eligible for population breast screening at the time of data collection. 73% reported high activity levels, 16% were current smokers and 12% had an alcohol problem (CAGE score > 2). A significantly lower proportion of smokers (74%) than non-smokers (87%) attended their last mammogram (p<0.001). There was a significant inverse relationship between number of cigarettes smoked/day, and mammogram attendance (p<0.001). In contrast, activity levels, BMI or an alcohol problem did not predict mammography attendance. Using the multivariable model to adjust for co-variates including socio-demographic, health and lifestyle variables, being a current smoker (OR 0.57, 95%CI 0.43-0.74), and smoking ≥20 cigarettes per day (OR 0.49, 95%CI 0.34-0.7), remained negatively associated with mammography attendance.

**Conclusion:** Smoking was the only lifestyle factor in our study associated with mammography attendance in Irish women eligible for population screening. Smoking is higher in lower socioeconomic groups, in whom screening uptake is poor. Smoking cessation advice and courses could potentially include information about breast screening, with complementary cancer prevention effect.

## O3.2 Incorporating adult learning theory into interventions to reduce recall rate in breast screening

### Shazia Khan, Gemma Smith, Anne-Marie Wason

#### **Correspondence:** Shazia Khan

##### Bradford Teaching Hospitals NHS Foundation Trust, Bradford, United Kingdom

**Background:** Demand on breast services has grown with increasing awareness of breast health and technological advances. However, given the workforce crisis, it is important that the current workforce is supported to work to and maintain high standards.

As part of Breast Screening Quality Assurance, we review our film reader statistics (FRQA) annually, including individual recall rates (RR). Over a number of years, it was noted that readers settled into 2 groups; one with a consistently low <5% RR and the second with higher RR (>5%) which did not decrease despite mentoring and regular interventions. Using adult educational learning theory, we reviewed the trends in reading practises for each group to identify ways to reduce the recall rate.

**Methods:** We identified that the low RR group led screening assessment clinics. In accordance with Kolb’s experiential learning cycle ^[1]^, to develop a skill there needs to be regular feedback and reflection to allow learners to refine their skills. We asked readers with a higher RR to identify, review and reflect on images that they recalled but were subsequently read normal by the 2nd & 3rd reader.

**Results:** Following establishment of this intervention, the overall RR for this group has fallen significantly and been maintained over 2 years. In the period 2015-2017 the average RR was 5.28/5.6 (1st/2nd reader). Following interventions the average RR over 2017-2019 was 4.7 and 3.9.

**Conclusion:** Reduction in RR was achieved and maintained by introducing an intervention incorporating adult learning theory.

[1] Kolb, D. A. Experiential Learning: Experience as the Source of Learning and Development. Prentice-Hall, Inc., Englewood Cliffs, N.J; 2014.

## O3.3 Male breast ultrasound: 2019 audit results

### Umit Aksoy Ozcan, Susan Williams, Marie Metelko

#### **Correspondence:** Susan Williams

##### The Shrewsbury and Telford Hospital NHS Trust, Shrewsbury, United Kingdom

**Background and Purpose:** Male breast cancer is rare whereas gynaecomastia is very common. Only asymmetrical gynaecomastia require breast imaging and focal lumps are amenable to clinical core biopsy. So the use of ultrasound in the assessment of male breast should be limited. The aim of this study is to audit the referral indications and ultrasound outcomes in male breast US (MBUS) patients against local guidelines.

**Methods:** In the last 5 years, 968 patients were referred for MBUS in our Trust. This audit includes the patients between 02/01/2019-04/12/2019. The duplicate patients and follow-ups were excluded from the study. In total, 197 patients were analysed (mean age: 58 (8-90) retrospectively. Referral diagnosis, age, US grading and clinical outcomes were noted.

**Results:** Of the 197 patients, 79% were gynecomastia (133), lipoma (21) or fat necrosis (2), and 15% (30) were normal. There was 1 chest wall lymphoma and 1 DCIS, and 9 (5%) patients had benign breast disease (fibroepithelial lesions, abscess, papilloma, sebaceous cysts, haematoma). In 122 patients (62%) clinical grade was not given, 66 had P2, 8 had P3, 1 had P5. 2 patients were scored as U4 and 4 patients as U3.

**Conclusions:** These results clearly show that 99% of the patients referred to MBUS were benign. And also 95% of the patients were clinically benign or not assessed. The excessive use of MBUS without a clinical indication leads to patient anxiety, increased waiting times and might delay the proper imaging to the patients who should have the priority in terms of clinical indication. Careful clinical assessment before ultrasound referral is mandatory for better care.

[1]. Best practice diagnostic guidelines for patients presenting with breast symptoms. NICE Nov 2010 https://www.evidence.nhs.uk/document?id=2013590&returnUrl=search%3Fq%3Dhc11%26sp%3Don&q=hc11

[2]. Shrewsbury and telford hospitals clinical guidelines for the management of breast cancer. June 2019

[3]. F. Draghi, C.C. Tarantino, L. Madonia Ultrasonography of the male breast J Ultrasound. 2011 Sep; 14(3): 122–129. . doi: 10.1016/j.jus.2011.06.004G. Ferrozzi

## O3.4 Is there a role for advanced practitioners to deliver biopsy results within breast screening?

### Joleen Kirsty Eden, Rita Borgen

#### **Correspondence:** Joleen Kirsty Eden

##### East Lancashire Hospitals NHS Trust, Burnley, United Kingdom

Qualitative research exploring the perceptions of Advanced Practitioner Radiographers (APRs) in delivering biopsy results within a single unit to NHS Breast Screening Programme (NHSBSP) assessment patients. A significant lack of published research in this area provides the rationale for this research, combined with an identified service-need and the increasing pressures on breast radiologists.^[1,2]^

**Method:** A grounded theory research design was used to interview six APRs individually in a single breast unit, followed by a focus-group with five of the APRs to acquire additional data and explore the identified themes using the Burnard constant comparative approach.^[3]^

**Results:** Five core themes emerged from the data; role of the APR, patient experience, efficiency, role boundaries and delivering results. The findings demonstrate the extension of the role is found to be within the scope of practice for APRs, providing they obtain the appropriate training and skills to deliver breast-biopsy results. Advanced practice experience is considered essential with the potential to benefit the radiography profession. Patient expectations were carefully considered along with communication difficulties. The SPIKES communication model is taught through NHS Connected Advanced Communication Skills Training and a requirement of the NHS Breast Screening Programme.^[4,5]^ There is potential to improve efficiency within the breast-screening service; however emotional impact requires consideration.

Effective implementation has successfully changed practice within this unit and may be adopted by other NHSBSP units to address service-need. Further guidance is needed from professional bodies.

Overall, with appropriate training and peer-support APRs feel able to effectively deliver results with a patient-centred approach.

[1]. Department of Health. ‘The NHS Cancer Plan’, 2000. pp. 1–98.

[2]. Royal College of Radiologists. Clinical radiology UK workforce census 2015 report.

[3]. Burnard P. A method of analysing interview transcripts in qualitative research. Nurse education today. 1991 Dec 1;11(6):461-6.

[4]. Baile WF, Buckman R, Lenzi R, Glober G, Beale EA, Kudelka AP. SPIKES—a six-step protocol for delivering bad news: application to the patient with cancer. The oncologist. 2000 Aug 1;5(4):302-11.

[5]. Public Health England. ‘Clinical guidance for breast cancer screening assessment (NHSBSP Publication No 49)’. 2016. pp. 1–36.

## P01 Physics QA and user QC for contrast-enhanced mammography in a multicentre study

### Nicholas Payne^1^, Oliver Morrish^2^, Fiona Gilbert^1^, Jennifer Oduko^2^

#### **Correspondence:** Nicholas Payne

##### ^1^University of Cambridge, Cambridge, United Kingdom; ^2^Cambridge University Hospitals NHS Foundation Trust, Cambridge, United Kingdom

The multi-centre, multi-modality, BRAID Trial includes an evaluation of the benefit of Contrast-Enhanced Mammography (CEM) for women with radiologically dense breasts. Imaging equipment used for breast cancer screening must undergo periodic testing to ensure that it is performing within expected parameters.

These tests are normally defined in guidance published by PHE and IPEM however these are not yet available for CEM. It was necessary therefore to develop a physics and user quality assurance procedure to monitor the CEM systems, ensuring that image quality and radiation dose performance remains constant throughout the study.

Following the spirit of the current national standards for screening [1-3] and informed by published research [4-5], quality assurance protocols were written for physics and user testing.

Both protocols outline measurements performed using a test object containing iodinated details of varying concentrations to assure that system is acquiring images with expected levels of signal-to-noise-ratio and detail conspicuity. For this purpose, medical physics teams at several of the study sites have purchased dedicated CEM phantoms (CIRS Inc., Norfolk, VA) to perform the QA while radiographers have been provided with laminated test objects created by Schofield et al.

While initially prescribed by the lead site, these QA and QC protocols are working documents to be improved upon over the course of the study based on feedback from local physics teams and the utility of the data collected. It is hoped that this work across multiple sites and vendors will inform CEM QA and QC protocols which can be adopted nationally.

[1] Kulama E, Burch A, Castellano I, Lawinski CP, Marshall N, Young KC. Commissioning and Routine Testing of Full Field Digital Mammography Systems (*NHSBSP Equipment Report 0604*). NHS Cancer Screening Programmes. 2009

[2] Moore AC, Dance DR, Evans DS, Lawinski CP, Pitcher EM, Rust A, Young KC. The Commissioning and Routine Testing of Mammographic X-ray Systems (*IPEM Report 89*). Institute of Physics and Engineering in Medicine. 2005

[3] Baxter G, Jones V, Milnes V, Oduko J, Philips V, Sellars S, Vegnuti Z. Routine Quality Control Tests for Full Field Digital Mammography Systems (*NHSBSP Equipment Report 1303 4th Ed.*). NHS Cancer Screening Programmes. 2013

[4] Oduko J, Homolka P, Jones V, Whitwam D. A Protocol for Quality Control Testing for Contrast-Enhanced Dual Energy Mammography Systems. *Breast Imaging: 12th International Workshop IWDM 2014 Gifu City, Japan 2014 Proceedings*. Heidelberg, Springer 2014 407-414

[5] Klausz R, Rouxel M, Mancardi X, Carton AK, Jeunehomme Patoureaus F. Introduction of a comprehensive phantom for the quality control of contrast enhanced spectral mammography. *European Congress of Radiology.* European Society of Radiology 2018 Poster C-2650

## P02 The Impact of contrast material on AEC performance and breast dose

### Kirsty Sherwin, Ryan Jones, Paul Connolly

#### **Correspondence:** Kirsty Sherwin

##### Integrated Radiological Services (IRS) Limited, Liverpool, United Kingdom

**Background:** Assessment of contrast-to-noise (CNR) and typical breast doses across a range of compressed breast thicknesses (CBT) are often performed simultaneously when assessing Automatic Exposure Control (AEC) performance. Assessment of CNR requires the presence of a 0.2mm aluminium insert used to generate contrast. However, presence of this insert can affect assessment of breast dose depending on the AEC operational design, particularly for thinner CBT. This paper presents the results of the impact of the presence aluminium on typical breast doses during AEC quality assurance (QA).

**Methods:** CNR and breast dose were assessed as part of AEC QA by testing with and without the Al insert under a range of automatic dose modes. Standard AEC testing methods described in NHSBSP0604[1] were performed on a range of systems from different manufacturers.

**Results:** For GE systems operating under CNT mode, assessed doses exceeded recommended remedial levels with the aluminium present but were below remedial levels without aluminium.

For Siemens systems, the presence of the aluminium made little difference to the doses observed with activation of AEC segmentation.

For Hologic systems, the aluminium affects doses only under Automatic AEC positioning. The presence of the aluminium has no impact on beam quality as the breast thickness independently selects beam quality.

**Conclusions:** Assessment of typical breast doses on GE systems should be performed without the aluminium insert. This allows for a more relevant comparison of typical breast dose across different systems.

[1]. **NHS Breast Screening Programme.** *Report 0604 - Commissioning and Routine Testing of Full Field Digital Mammography Systems.* Sheffield: NHS Cancer Screening Programmes, Apr 2009.

## P03 "Real world" radiomics of breast cancer from multi-vendor MRI studies for prediction of nodal status and disease survival

### Simon Doran^1^, Santosh Kumar^1^, Matthew Orton^1^, James d'Arcy^1^, Fenna Kwaks^1^, Elizabeth O'Flynn^2^, Kate Downey^3^, Mitch Dowsett^1^, Nicholas Turner^1^, Dow-Mu Koh^1^

#### **Correspondence:** Elizabeth O'Flynn

##### ^1^Institute of Cancer Research and The Royal Marsden Hospital, London, United Kingdom; ^2^St George's University Hospitals NHS Foundation Trust,, London, United Kingdom; ^3^The Royal Marsden Hospital, London, United Kingdom

**Background:** Most MR radiomics studies to date have used "pure" datasets accrued from single-vendor, single-field-strength scanners. This does not reflect the ultimate generalisability of AI models and there is no way of determining how such models will be for input data from different sources.

**Methods:** 156 patients with pathologically proven breast cancer underwent multi-contrast MRI prior to neoadjuvant chemotherapy and/or surgery. From these, 92 patients were identified for whom T2-weighted, diffusion-weighted and contrast-enhanced T1-weighted sequences and clinicopathological variables were available. Regions of interest were drawn on the images, and from these semantic and calculated radiomics features were derived. Model fits were generated for four different types of classification model (support vector machine, random forest, extreme gradient boosting and naïve Bayes) to predict pathological nodal status. In parallel work, survival modelling was performed using random survival forests.

**Results:** Prediction of nodal status yielded AUC values of 0.74 (95% CI [0.71, 0.78]) for clinical variables alone, 0.69 (95% CI [0.65, 0.72]) for radiomic features only, and 0.79 (95% CI [0.75, 0.82]) for radiomics and clinical features together. Prediction of scanner platform from the radiomics features yielded extremely high values of AUC between 0.86 and 1 for the different classes examined. Survival analysis, gave out-of-bag prediction errors of 18.1% (clinical features only), 33.5 – 39.7% (radiomic features from different combinations of image contrasts) and 24.4 – 33.8% (clinical plus radiomics features).

**Conclusions**: Potentially useful radiomics signatures for disease classification may be derived from heterogeneous “real world” input data, despite clear confounding information being present.

[1]. Papanikolaou, N., C. Matos, and D.M. Koh, *How to develop a meaningful radiomic signature for clinical use in oncologic patients.* Cancer Imaging, 2020. **20**: p. 1-10.

[2]. Zwanenburg, A., et al., *The image biomarker standardization initiative: standardized quantitative radiomics for high-throughput image-based phenotyping.*Radiology, 2020. **295**(2): p. 328-338.

## P04 Investigation of automated breast lesion classification based on focused RF frequency spectra

### Christian Graff, Laura Glaubes, Trushali Doshi, David Gibbins, Louis Tsui

#### **Correspondence:** Christian Graff

##### Micrima Ltd, Bristol, United Kingdom

**Background**: The Micrima MARIA® system is an ultra-wideband (3-8GHz) radar imaging system sensitive to electromagnetic permittivity contrast in breast tissue. Using a hemispherical array of 60 antennas, 1770 channels are measured over 101 frequencies and focused to form a 3D image of scattering. This phantom study investigates the feasibility of focused spectra for classification of lesions.

**Methods**: Two lesion classes were included; benign cyst, modelled as water-filled glass spheres (diameter = 1-2 cm, relative permittivity ε_r_ = 78 @ 3GHz) and malignant lesion, modelled as spiculated ovoids consisting of polyurethane, graphite, and carbon black (diameter = 1-2 cm, ε_r_ = 29 @ 3GHz). Lesions were imaged on two MARIA® systems while embedded in an analogue of adipose breast tissue (liquid paraffin and water emulsion) at multiple locations in 3 breast volumes (460cc, 700cc, and 1000cc). In total 288 images were acquired. Focused spectra from lesion ROIs were analysed to assess classification performance.

**Results**: Multivariate analysis of variance of 10 frequency bands indicated statistically significant effect of lesion class, Pr(>F) <2.2e-16, with strongest effect for the 3-3.5GHz band. Leave-one-out cross-validation of ROI classification based on thresholding the mean value of this band achieved sensitivity 0.75 (0.67-0.82) and specificity 0.79 (0.71-0.85).

**Conclusions**: Results indicate that focused spectra provided by MARIA®, particularly in the 3-3.5GHz band, are sensitive to differences in breast lesion characteristics associated with the likelihood of malignancy in vivo. The response in this frequency band when used as a decision variable yields promising classification performance, warranting further clinical investigation.

## P05 Coding accuracy following the introduction of electronic coding: a single-centre experience from a breast imaging department

### Anita Bolina^1^, Kathy Edmonds^2^, Neil Upadhyay^2^

#### **Correspondence:** Anita Bolina

##### ^1^Imperial College London, London, United Kingdom; ^2^Imperial College Healthcare NHS Trust, London, United Kingdom

**Background:** Accurate coding of procedures is essential for reimbursement within radiology departments. Under a new system within the Imperial College Healthcare NHS Trust, income will soon only be generated if procedures are coded within the Cerner© electronic patient record (EPR), with radiologists and advanced practitioners responsible for inputting the code. However, issues related to familiarity with EPR and time pressures within clinics can affect the accuracy of coding. The aims of this study were to investigate the rate of non-coding for biopsy procedures, and subsequently how much income would be lost through non-coding.

**Methods:** All biopsies performed during a sample month of August 2019 were identified using the weekly MDT lists, which captures all specimens sent to histopathology. Ultrasound guided core biopsies were included, and the EPR was checked for coding of the biopsy. Coding accuracy was cross-checked against the Radiology Information System (RIS).

**Results:** 97 ultrasound guided core biopsies were included in the study. 7.2% were axilla biopsies, 3.1% biopsied both breast and axilla, and 89.7% were breast biopsies. Only 16.5% of biopsies were coded on EPR. Cost analysis revealed £11,826 loss of income in one month due to procedural non-coding. Income gained from biopsy coding was £2336.

**Conclusions:** Overall a large volume of income was lost through non-coding. Many radiologists find the current method of electronic coding cumbersome and time-consuming during clinics, which is likely contributing to low coding rates. An automated system linking RIS entries to EPR would generate a huge cost saving for the department.

## P08 Early results from BreastCheck age extension in the Republic of Ireland

### Patricia Fitzpatrick^1^, Therese Mooney^1^, Helen Byrne^1^, Lorraine Fahy^1^, Fidelma Flanagan^2^, Alissa Connors^2^, Aideen Larke^2^, Ann O'Doherty^2^

#### **Correspondence:** Patricia Fitzpatrick

##### ^1^National Screening Service, Dublin, Ireland; ^2^BreastCheck, Dublin, Ireland

BreastCheck is the national breast screening programme in the Republic of Ireland. In late 2015, age-range extension was launched for women ≥65 years. A small number of these women had never been invited before, perhaps due to immigration/return to Ireland or recent self-registration. However, the majority had previously been invited for screening so the age extension resulted in a higher number of subsequent and a small number of initial women ≥65 being invited.

Data is routinely collected on all women invited and screened through BreastCheck ^[1]^. The current age range extension is being rolled out on a phased basis. By 2021, all women aged 50-69 will be invited.

In 2017-18 608 women aged 65+ were invited for the first time, with 87 women screened (14.7%). 19,615 older women were invited for subsequent screening, and 16,650 attended (84.9%). Among these 16,650, the recall rate was similar to women aged 55-64; the benign biopsy rate (BOBR) was lower than for any other age groups, while the cancer detection rate (CDR) was higher (Table 1).

**Table 1:** **(abstract P08). See text for description.**

**Performance parameter**	**50-54 years**	**55-59 years**	**60-64 years**	**65+ years**
Recalled to assessment	3.4%	2.8%	2.7	2.8
BOB rate*	1.06	0.65	0.71	0.42
CDR*	5.55	5.14	6.75	7.09

* Per 1,000 women screened

Early results show good uptake and, as anticipated, higher CDR and lower benign biopsy rate among women in the age extension. As BreastCheck has a two year interval, CDR is lower than the corresponding NHS figures with a three-year interval.^[2]^

1. BreastCheck. BreastCheck Annual Report 2017-2018 [Internet]. Available from: https://www.breastcheck.ie/sites/default/files/bcheck/documents/breastcheck-programme-report-2017-2018-final-060120.pdf (accessed Dec 2019)
2. NHS Digital. Breast Screening Programme England 2018-2019. [Internet]. Available from: https://files.digital.nhs.uk/0A/9D9F34/breast-screening-programme-eng-2018-19-report.pdf (accessed Dec 2019)

## P09 Does yearly mammographic surveillance put a large group of younger breast cancer patients at further risk?

### Heather V Mower^1^, Desiree O'Leary^2^

#### **Correspondence:** Heather V Mower

##### ^1^Northern Devon Healthcare NHS Trust, Barnstaple, United Kingdom; ^2^University of Keele, Keele, United Kingdom

**Background and Objectives**: National^[1,2]^ and international guidelines^[3-7]^ require women diagnosed with breast cancer at an early age to undergo numerous annual mammograms beyond diagnosis until screening age. This despite younger women having dense breast tissue^[8]^ with reduced mammographic sensitivity for detection of abnormalities^[3]^, and the lifetime risk for developing radiation-induced cancer being highest in younger women^[9]^. A current UK trial (Mammo-50^[10]^) investigates targeted surveillance for patients over 50 years, but there is no known trial seeking change for younger women. Ultimately: Could there be a targeted approach for follow-up of younger age women breast cancer groups? This study aims to investigate how many younger women are affected by non-targeted screening.

**Methods:** Younger women under 45 years who have undergone more than 5 years of annual mammograms are investigated for: grade of cancer diagnosis, family history, breast tissue density, whether original cancer was mammographically occult, number of mammograms since diagnosis, and whether discharged from annual surveillance (including reason).

**Results:** UK-wide results suggests 9% of new breast cancers were diagnosed in women below 44years in 2014-2016 ^[11]^. Sensitivity of surveillance mammography for detection of recurrence was 64-67% with a specificity from 85-97%^3^, while sensitivity was reduced in patients with increased mammographic breast density to ~30%^9^. This study seeks to determine the current accuracy of these figures.

**Conclusions:** Early results suggest that this is a rising population of breast cancer patients**.** The ultimate aim of the study is to determine whether a more suitable mammographic surveillance can be determined for this group of younger women.

1. The Royal College of Radiologists (RCR). *Guidance on screening and symptomatic breast imaging.* 4th edition. 2019. Available from:  https://www.rcr.ac.uk/system/files/publication/field_publication_files/bfcr199-guidance-on-screening-and-symptomatic-breast-imaging.pdf [Accessed 19th December 2019].
2. National Institute for Health and Care Excellence (NICE). *Early and locally advanced breast cancer: diagnosis and management, [NG101].* 2018. Available from: https://www.nice.org.uk/guidance/ng101 [Accessed 19th December 2019].
3. Robertson C, Arco Ragupathy SK, Boachie C, Dixon JM, Fraser C, Hernández R, et al. The clinical effectiveness and cost-effectiveness of different surveillance mammography regimens after the treatment for primary breast cancer: systematic reviews registry database analyses and economic evaluation. *Health Technology Assessments.* 2011;15(34):1-322. Available from: doi:10.3310/hta15340.
4. Runowicz CD, Leach CR, Henry NL, Henry KS, Mackey HT, Cowens-Alvarado RL, et al. American Cancer Society/American Society of Clinical Oncology Breast Cancer Survivorship Care Guideline. *Journal of Clinical Oncology*. 2016;34(6):611–635. Available from: doi:10.1200/JCO.2015.64.3809.
5. National Comprehensive Cancer Network (NCCN). *Clinical practice guidelines in oncology: breast cancer, version 3*. 2019. Available from: https://www.nccn.org/professionals/physician_gls/pdf/breast.pdf [Accessed 24th December 2019].
6. American College of Radiology (ACR). *ACR Appropriateness Criteria® Stage I Breast Cancer: Initial Workup and Surveillance for Local Recurrence and Distant Metastases in Asymptomatic Women,* 2019. Available from : https://acsearch.acr.org/69496/Narrative/ [Accessed 24th December 2019].
7. Cardoso F, Kyriakides S, Ohno S, Penault-Llorca F, Poortmans P, Rubio IT, et. al. on behalf of the ESMO Guidelines Committee. Early breast cancer: ESMO Clinical Practice Guidelines for diagnosis, treatment and follow-up. *Annals of Oncology.* 2019;30(8):1194–1220. Available from: doi.org/10.1093/annonc/mdz173.
8. Freer PE. Mammographic breast density: Impact on breast cancer risk and implications for screening. *Radiographics.* 2015;35(2):302-315. Available from: doi:10.1148/rg.352140106.
9. Warren LM, Dance DR, Young KC. Radiation risk of breast screening in England with digital mammography, *British Institute of Radiology.* 2016;89(1067). Available from: doi:10.1259/bjr.20150897.
10. Current Controlled Trials. Warwick: Clinical Trials Unit. ISRCTN48534559. *Mammo-50: Mammographic surveillance in breast cancer patients aged 50 years and over.* First received 2013. Available from: 10.1186/ISRCTN48534559.
11. Cancer Research UK. *Breast cancer incidence (invasive) statistics.* Available from: https://www.cancerresearchuk.org/health-professional/cancer-statistics/statistics-by-cancer-type/breast-cancer/incidence-invasive#heading-One [Accessed 19th December 2019].

## P10 A feasibility cohort study to determine the role of MRI in guiding peri-operative decision making for chest wall perforator flap breast reconstruction

### Anna Heeney, Rachel O'Connell, Nihal Gonen, Lynsey Williams, Katherine Krupa, Edward StJohn, Jennifer Rusby, Steven Allen, Peter Barry

#### **Correspondence:** Anna Heeney

##### The Royal Marsden Hospital, London, United Kingdom

**Background**: Chest wall perforator flaps (CWPFs) are a volume replacement technique which permits breast conserving surgery in selected cancer patients where mastectomy is the only alternative^[1]^. CWPFs are predicated on a blood supply from the lateral chest wall behind the breast. These flaps are based on a perforating artery which courses through the soft tissue into the sub-dermal plexus to perfuse the tissue. Examples includes the lateral intercostal and Lateral Thoracic artery perforators (LICAP and LTAP respectively) ^[2]^. The origin, distribution and calibre of these perforator vessels is mapped both pre-operatively and intra-operatively using hand-held acoustic doppler but can be ambiguous. We therefore aim to determine the potential role of MRI in mapping the vascular anatomy to aid surgical planning.

**Methods**: We retrospectively reviewed our entire cohort of breast cancer patients who had CWPF surgery. We conducted radiological review of breast MRI performed as part of routine pre-operative imaging to determine perforator anatomy.

**Results**: Of 43 patients who underwent CWPFs, 22 (51%) had MRI imaging of their breasts prior to surgery. Evidence of perforator vessels on MRI were noted and formal analysis of our results will be completed and presented in full at the symposium.

**Conclusion**: Although cross sectional imaging is not a pre-requisite for performing CWPFs a significant proportion of breast cancer patients already undergo breast MRI for oncological purposes. MRI may provide useful additional information regarding the origin, location and calibre of perforator vessels to aid in surgical planning for these flaps.

[1]. Soumian S, Parmeshwar R, Chandarana M, Marla S, Narayanan S, Shetty G. Chest wall perforator flaps for partial breast reconstruction: Surgical outcomes from a multicenter study. Arch Plast Surg. 2020;47(2):153-9.

[2]. McCulley SJ, Schaverien MV, Tan VK, Macmillan RD. Lateral thoracic artery perforator (LTAP) flap in partial breast reconstruction. J Plast Reconstr Aesthet Surg. 2015;68(5):686-91

## P12 The wire-free breast localisation system (radiofrequency tag): is this the beginning of the end for wires

### Maha Buhleigah, Alexandra Christou, Rathinasabapathy Rathinaezhil, Samy Shaheed, Alexandros Mytafidis, Katerina Lekanidi, Rebecca Locke, Charles Zammit

#### **Correspondence:** Maha Buhleigah

##### Brighton and Sussex University Hospitals NHS Trust, Brighton, United Kingdom

**Purpose:** To share our unit experience in using the Radiofrequency Tag, (RFT) with a view to replace the conventional wire localisation and improve patients’ preoperative experience.

**Methods:** From August 2019 to January 2020, 12 patients with 13 non-palpable breast lesions (one was bilateral) underwent preoperative lesion localisation using the RFT (LOCalizer™). The device consists of a small radioactive free tag with unique number and a polypropylene cap to prevent migration, a 12gauge needle applicator, a radiofrequency reader and surgical reader probe, which is an 8mm pencil size allowing for small incisions.

**Results:** 10/13 lesions were found to be invasive cancers, 1/13 DCIS and 2/13 B3 lesions. 46.2% were micro-calcifications and 53.8% were masses with average mammographic size of 11.5mm (5-20mm). The tag was placed satisfactorily in 12/13 lesions. For 1 lesion a second tag had to be inserted due to misplacement of first one. 11/13 lesions underwent wide local excision and 2/13 excision biopsies. 3 intraoperative re-excisions were done in 2 patients. The average weight of the specimen was 38.75. Average time of operation was 17minutes (4-35 minutes). Average ease of operation was 4.7 (3 to 6). Only 1 patient needed re-excision on a later day (7.8%). The average time between localisation and operation was 2.2 days (0-6 days).

**Conclusions:** Despite small number of cases, RFT is an accurate, easy and wire free technique which can be inserted up to one month prior to surgery and can potentially be used as an alternative to wires and improve patients’ experience.

## P13 Complication rates following vacuum biopsy and implications for lesion localisation

### Dianne Lennox, Nerys Forester

#### **Correspondence:** Dianne Lennox

##### Newcastle Hospitals, Newcastle, United Kingdom

**Background:** Despite the increasing use of vacuum assisted biopsy in both first and second line breast biopsies, little is known about the true complication rate of this procedure, and whether it differs between 10 and 7G biopsies, or if any complications impact on subsequent patient management.

**Methods:** Review of all stereotactic 7/10G biopsies between 01/04/18 and 31/03/19 performed as part of screening assessment in a single centre. This comprised both review of patient notes and images from the time of biopsy. Complications documented included bleeding/haematoma formation, pain and inaccurate clip placement.

**Results:** 670 first and second line stereotactic procedures were performed in one year. The rate of immediate complications in both groups was low. No serious complications were documented. The rate of complications affecting surgical planning was minimal.

**Conclusion:** Complications from vacuum biopsy are rare. However, occasionally immediate complications such as haematoma formation occurred which made surgical planning less straightforward, or clip placement/migration occurred which required innovative localisation methods to allow onward patient management. These cases will be illustrated and discussed. It was very difficult to measure delayed complications such as infection, although collectively we are only aware of one case of post biopsy infection occurring over the past few years.

## P14 Abbreviated breast MRI (FAST MRI): A multi-centre reader-training study for NHS Breast Screening Programme (NHSBSP) multi-professional mammogram readers (funded by the National Institute for Health Research (NIHR) Research for Patient Benefit (RfPB) funding stream (ISRCTN 16624917)

### Lyn Jones^1^, Rebecca Geach^1^, Sam Harding^1^, Andrea Marshall^2^, Sadie McKeown-Keegan^1^, Sian Taylor-Phillips^3^, Premkumar Elangovan^4^, Sarah Vinnicombe^5^, Elizabeth O'Flynn^6^, Janet Dunn^2^

#### **Correspondence:** Lyn Jones

##### ^1^North Bristol NHS Trust, Bristol, United Kingdom; ^2^Warwick Clinical Trials Unit at the University of Warwick, Warwick, United Kingdom; ^3^University of Warwick Medical School, Warwick, United Kingdom; ^4^Royal Surrey County Hospital NHS Foundation Trust, Guildford, United Kingdom; ^5^Gloucestershire Hospitals NHS Foundation Trust, Gloucester, United Kingdom; ^6^St George's University Hospitals NHS Foundation Trust, London, United Kingdom

**Background:** To validate the feasibility of training mammogram readers in FAST MRI interpretation ^[1]^ we developed display software (MedXViewer) containing ground truth information for each FAST MRI case to train and assess mammogram readers from NHSBSP sites.

**Methods:** A per breast analysis of the frequency of the results against the true outcome was obtained overall and for each reader. Differences in accuracy, sensitivity and specificity across reader groups (group 1 = mammogram readers experienced in breast MRI interpretation; group 2 = mammogram readers with no previous experience in breast MRI interpretation) were analysed using a multilevel generalised mixed model to account for multiple readers per case.

**Results:** 37 NHSBSP mammogram readers (17 in group 1 and 20 in group 2) attended the training day (6 NHSBSP sites) and completed the reading task of 125 cases (250 breasts) (total=9250 reads).

The 83% accuracy achieved by Group 2 (4129/5000 (95% CI 82-84%)) was significantly lower than that by Group 1 (3814/4250 (90%; 89-91%); p<0.0001) but differed by only 7%.

Accuracy improved for the group 2 readers from the first 55 cases to the remaining 70 cases (p=0.02), whereas there was no significant improvement for the expert readers of group 1 (p=0.81).

**Conclusions**: This study validates the feasibility of training mammogram readers to interpret FAST MRI with only one day of training. Improvement in performance with experience by the group 2 readers indicates a learning curve and suggests the performance gap between the two groups might be narrowed by further training.

[1] Jones L, Geach R, Harding S et al. Can mammogram readers swiftly and effectively learn to interpret first post-contrast acquisition subtracted (FAST) MRI, a type of abbreviated breast MRI?: a single centre data-interpretation study. The British Journal of Radiology 2019;92:20190663 doi: 10.1259/bjr.20190663

## P15 Molecular breast imaging: A qualitative study

### Helen Elliott^1^, Alison Bray^1^, Sara Graziadio^2^, Timothy Powell^1^, Nerys Forester^3^

#### **Correspondence:** Helen Elliott

##### ^1^Newcastle upon Tyne Hospitals NHS Foundation Trust; Translational and Clinical Research Institute, Newcastle University, Newcastle, United Kingdom; ^2^NIHR Newcastle In Vitro Diagnostics Co-operative; Newcastle upon Tyne Hospitals NHS Foundation Trust, Newcastle, United Kingdom; ^3^Newcastle upon Tyne Hospitals NHS Foundation Trust, Newcastle, United Kingdom

**Background**: Breast cancer screening using mammography has poorer sensitivity in dense tissue.^[1,2]^ Retrospective studies have shown higher sensitivity of Molecular Breast Imaging (MBI) in this group.^[3,4]^ UK stakeholder perceptions of MBI are unknown and key to understanding the value of, and route to, adoption.

**Method**: Semi-structured interviews with stakeholders (radiologists, technologists, policy-makers) around:

- NHS Breast Cancer Screening pathway
- new MBI pathway(s)
- routes for MBI adoption

**Results**: Thematic analysis of ten interviews, using NVivo, identified six themes:

1. Scan time (40min) and radiation dose (740MBq) are inversely proportional. Reducing both is important, but time appears to be the bigger barrier to implementation.
2. An MBI biopsy function could be valuable.
3. Mobile MBI services seem unfeasible. Although nuclear medicine scans could be implemented in mobile units, maintaining safety would be expensive.
4. Location, staffing, and radiotracer management require careful planning**.** Preparation of tracers requires specialist radiopharmacy facilities. The skills and expertise of both breast screening (for breast positioning) and nuclear medicine (for image acquisition) staff are important.
5. Diagnostic interpretation of MBI images (automated by contrast-to-noise ratio, and/or visually by radiologists), and minimum image resolution (for identification of the smallest tumours) must be established and validated.
6. MBI is highly unlikely to replace mammography, but may be valuable for:
  screening woman with dense breasts/implants/at higher risk of breast cancer (as an MRI alternative),
  monitoring disease response during neo-adjuvant chemotherapy,
  post-cancer surveillance in dense breasts.

**Conclusion**: Evidence indicates a potential place for MBI for certain groups in the UK.

[1] Kolb TM, Lichy J, Newhouse JH. Comparison of the performance of screening mammography, physical examination, and breast US and evaluation of factors that influence them: an analysis of 27,825 patient evaluations. Radiology. 2002;225:165–75.

[2] Rosenberg RD, Hunt WC, Williamson MR, Gilliland FD, Wiest PW, Kelsey CA. Effects of age, breast density, ethnicity, and estrogen replacement therapy on screening mammographic sensitivity and cancer stage at diagnosis: review of 183,134 screening mammograms in Albuquerque, New Mexico. Radiology. 1998;209:511–8.

[3] Kim BS, Moon BI, Cha ES. A comparative study of breast-specific gamma imaging with conventional imaging modality in breast cancer patients with dense breasts. Ann Nucl Med. 2012 Dec;26(10):823-9

[4] Rhodes DJ, Hruska CB, Conners AL, Tortorelli CL, Maxwell RW, Jones KN, et al. Molecular Breast Imaging at Reduced Radiation Dose for Supplemental Screening in Mammographically Dense Breasts. AJR Am J Roentgenol. 2015 Feb;204(2):241–51.

## P16 A pilot study to examine the value of opportunistic mammography in women aged 35-49 years in a symptomatic setting

### Michelle Boyce, Penelope Moyle

#### **Correspondence:** Michelle Boyce

##### Cambridge Breast Unit, Cambridge, United Kingdom

**Background:** There is a lack of UK guidelines regarding the most appropriate age for symptomatic mammography ^[1,2]^, many centres begin at >40 years, others at >35 years. ‘Best Practice’ guidelines state mammography isn’t indicated in a majority of patients <40 years ^[3]^ so if a clinical breast examination (CBE) is normal/benign, mammography would be ‘opportunistic’. Previous studies debated the age for opportunistic mammography considering radiation protection, and risks of missing breast cancers (BCa) in younger women ^[4].^

**Objectives**:

1. Investigate how many women 35-49 years had an E1,E2orE3 CBE, subsequently diagnosed with BCa following mammography.
2. Evaluate the value of mammography in women 35-49years.
3. Determine whether setting the age at >40-years would reduce radiation without missing BCa’s in women 35-39years.

**Method:** Inclusion: Women 35-49years old with E1, E2 and E3 CBE scores. Excluded: male patients, <35 and >49 years, high-risk gene carriers, previous BCa’s and CBE scores of E4/E5. Cohort was 3614 women attending the breast clinic during 2016/2017. No additional imaging was performed, no symptomatic pathways were affected.

Eligible participants were categorised into sub age-groups

35-39 years (n=1126)

40-44 years (n=1292)

45-49 years (n=1196)

CBE scores, Imaging scores, histopathology results were directly compared between the age groups.

**Conclusions:** Regardless of CBE or mammography scores, all ultrasound examinations were scored U3 or above prompting biopsies, therefore not one cancer was missed/diagnosis delayed.

The threshold for mammography could be set at >40 years in the event of a normal or benign CBE without missing BCa’s, simultaneously reducing radiation exposure.

[1] Antman K, Shea, S. Screening mammography under age 50. JAMA. 1999 April 28; 281(16): 1470-1472. Available from: https://jamanetwork.com/journals/jama/fullarticle/773467 (accessed June 2020)

[2] Britton P, Duffy SW, Sinnatamby R, Wallis MG, Barter S, Gaskarth M, O'Neill A, Caldas C, Brenton JD, Forouhi P, Wishart GC. One-Stop diagnostic breast clinics: How often are breast cancers missed? BJC. 2009 May 19; 100(12): 1873-8. Available from: 10.1038/sj.bjc.6605082 (accessed June 2020)

[3] Willett AM, Michell MJ, Lee MJR (eds). Best practice guidelines for patients presenting with breast symptoms. London Department of Health, 2010 November. p12. Available from: https://associationofbreastsurgery.org.uk/media/1416/best-practice-diagnostic-guidelines-for-patients-presenting-with-breast-symptoms.pdf (accessed June 2020)

[4] Buckley A, Healy N, Quinn A, O’Keefe S.A. The value of routine screening mammography in women aged 35-39 years in a symptomatic breast unit. Clin Rad. 2017 June; 72(6): 517.e7-517.e12. Available from: 10.1016/j.crad.2016.12.007 (accessed June 2020)

## P17 Breast screening reporting and arbitration - current picture and future possibilities

### Lisa Hackney^1^, Ala Szczepura^2^, Derek Renshaw^2^, Louise Moody^2^, Becky Whiteman^3^

#### **Correspondence:** Lisa Hackney

##### ^1^Macclesfield District General Hospital, Macclesfield, United Kingdom; ^2^Coventry University, Coventry, United Kingdom; ^3^Baxter Healthcare, Compton, United Kingdom

**Purpose/Background/Objectives**: To explore variation in current reporting and decision-making strategies nationally for recalls within breast screening services. To seek to correlate findings with unit performance, based on specific criteria from published national service data (KC62), and produce recommendations for future effective use of arbitration processes. To explore the role of Artificial Intelligence (AI) in this setting.

**Methods**: A mixed-method, explanatory sequential study with methodological integration of two national surveys (49 Units), qualitative interviews with professionals in varying roles (n=21), and specific performance metrics (KC62). Thematic analysis, including triangulation to compare and contrast data.

**Results**: Four main themes were identified: (1) Service Variation (2) Culture/implementation climate (3) Planning the service/standardisation (4) Task shifting and PHE arbitration guidance (5) Artificial Intelligence

**Conclusions**

Key recommendations:

1: Blinded double reading recommended to obtain the best insight into individual reader performance and standardisation of practice.

2: Blinded arbitration (anonymisation of the reporter) - to obtain independent non-biased opinions.

3: Careful selection of arbitrators - BSIS data to support delegation of solitary third reader arbitration/consensus leads.

4: NBSS updates -to improve usability, enable optimisation of consensus groups and to facilitate true blind reading/arbitration.

5: Consider centralisation/independent arbitration (but internal to the NHSBSP) - may normalise arbitration and provide sufficient arbitrations per individual to allow more accurate performance monitoring.

6: AI could potentially tackle some of the current challenges in breast screening, including improved accuracy of detection, increased efficiency, and advance detection of early cancers.^[1,2,3]^ Further research is needed on optimising human/AI decision-making.

[1] Trister, A.D., Buist, D.S.M., and Lee, C.I. (2017) ‘Will Machine Learning Tip the Balance in Breast Cancer Screening?’ *JAMA Oncology* 3 (11), 1463.

[2] Rodriguez-Ruiz, A., Lång, K., Gubern-Merida, A., Broeders, M., Gennaro, G., Clauser, P., Helbich, T.H., Chevalier, M., Tan, T., Mertelmeier, T., Wallis, M.G., Andersson, I., Zackrisson, S., Mann, R.M., and Sechopoulos, I. (2019) ‘Stand-Alone Artificial Intelligence for Breast Cancer Detection in Mammography: Comparison With 101 Radiologists’. *Journal of the National Cancer Institute* 111 (9), 916–922.

[3] McKinney, S.M., Sieniek, M., Godbole, V., Godwin, J., Antropova, N., Ashrafian, H., Back, T., Chesus, M., Corrado, G.C., Darzi, A., Etemadi, M., Garcia-Vicente, F., Gilbert, F.J., Halling-Brown, M., Hassabis, D., Jansen, S., Karthikesalingam, A., Kelly, C.J., King, D., Ledsam, J.R., Melnick, D., Mostofi, H., Peng, L., Reicher, J.J., Romera-Paredes, B., Sidebottom, R., Suleyman, M., Tse, D., Young, K.C., De Fauw, J., and Shetty, S. (2020) ‘International Evaluation of an AI System for Breast Cancer Screening’. *Nature* 577 (7788), 89–94.

## P18 Feasibility of training NHS breast screening programme (NHSBSP) mammogram readers to interpret abbreviated breast MRI (FAST MRI)

### Lyn Jones^1^, Rebecca Geach^1^, Sam Harding^1^, Jennifer Wookey^1^, Andrea Marshall^2^, Sarah Vinnicombe^3^, Claire Hulme^4^, Sian Taylor-Phillips^2^, Sadie McKeown-Keegan^5^, Janet Dunn^2^

#### **Correspondence:** Lyn Jones

##### ^1^North Bristol NHS Trust, Bristol, United Kingdom; ^2^University of Warwick, Warwick, United Kingdom; ^3^Gloucestershire Hospitals NHS Foundation Trust, Gloucester, United Kingdom; ^4^University of Exeter, Exeter, United Kingdom; ^5^North Bristol Hospital NHS Trust, Bristol, United Kingdom

**Background**: Early diagnosis of breast cancer (BC) saves lives and is the aim of the NHSBSP, but screening with mammograms fails to prevent some stage 2-4 BCs (underdiagnosis), which often present symptomatically between screening rounds (interval cancers). Breast MRI is the gold standard but its high cost prevents its use in breast cancer screening for all but those at highest risk. A type of abbreviated breast MRI called FAST MRI can identify aggressive breast cancers, and its short acquisition and reading times promise potential cost effectiveness.

**Aims and objectives**: To pilot an electronic training programme for FAST MRI within the NHSBSP workforce.

**Methods**: A standardised teaching tool for FAST MRI and a data collection tool, which includes a dataset of 250 case images with known outcome, has been developed as an electronic training programme. The resultant electronic teaching device, following validation, is being piloted across six NHSBSP screening units in England.

**Results**: After half a day’s structured training within a single centre, NHSBSP mammogram readers, without previous experience of breast MRI interpretation, showed good accuracy of 85% (95% confidence interval 82-87%). Similar or better accuracy is expected with the electronic training package.

**Conclusion**: The electronic training package will be rolled out across the UK to support a multi-centre study, which will potentially enable women at moderate risk of developing breast cancer to be screened with FAST MRI within the NHSBSP as part of stratified/personalised screening to identify biologically-relevant breast cancers that would otherwise have been missed by mammogram.

## P19 The development of a standardised positioning and compression protocol for use within UK breast screening and symptomatic services

### Muniratu Osmanu, Claire Mercer, John Thompson, Katy Szczepura

#### **Correspondence:** Muniratu Osmanu

##### University of Salford, Salford, United Kingdom

**Background:** Mammography is associated with pain/discomfort and this is mainly due to positioning and the compression applied to the breast. ^[2]^ The aim of the research is to develop an evidence-based protocol that may help reduce pain/discomfort. The angle of image receptor (IR) on the mediolateral oblique (MLO) projection plays a vital role in the distribution of pressure through the breast. When the IR angle is perpendicular to the sternum during compression, there should be an even pressure balance and increased breast coverage. ^[1]^

**Method:** A phantom study was conducted on a model torso with breast attachment. A digital inclinometer was used to take the angle of model’s sternum before it was positioned for MLO. Xsensor pressure mat was secured to the surfaces of the compression paddle and IR to read and record pressure distribution applied on the breast phantom. Compression of 10daN was applied to breast phantom and pressure readings and breast foot print were recorded with the IR at various angles in the multiples of 5 from 40^0^ to 70^0^. Numerical pressure data recorded on the mat was transferred onto excel and analysed.

**Results:** IR angles at 55^0^ to 65^0^ produced a more even pressure and area balance. The recorded sternal angle of model was 60^0^.Conclusion: When the IR angle is parallel or close to the angle of the sternum, there is an even distribution of pressure and area. A study in human female volunteers using this method is in progress.

[1]. Hogg, P. P. e., Kelly, J. e., & Mercer, C. e. (2015). Digital mammography: a holistic approach. In: Cham, Switzerland: Springer.

[2]. Papas, M. A., & Klassen, A. C. (2005). Pain and discomfort associated with mammography among urban low-income African-American women. Journal of Community Health, 30(4), 253-267. doi:10.1007/s10900-005-3704-5

## P20 Socio-demographic, lifestyle and health-related factors associated with breast self-examination in women aged 50 to 64 years in Ireland

### Nancy Bhardwaj^1^, Patricia Fitzpatrick^2^

#### **Correspondence:** Nancy Bhardwaj

##### ^1^University College Dublin, Dublin, Ireland; ^2^St Vincent's University Hospital and University College Dublin, Dublin, Ireland

**Background:** Regular breast self-examination (BSE) is a simple, cost-effective way of early detection of breast cancer, particularly for women outside the eligible age bracket for mammography and for interval cancers.^[1]^ BSE is routinely recommended but compliance is generally lower than desired and highly variable.^[1, 2]^ The aim of this study was to identify socio-demographic, lifestyle and health-related factors associated with BSE practice among women in Ireland.

**Methods:** The Irish Longitudinal Study of Ageing (TILDA) wave 4 was used; TILDA collects information on all aspects of health, economic and social circumstances from people aged 50 and over in a series of data collection waves once every two years. The subgroup of women aged 50-64 (eligible breast screening age in 2016) was selected. Following univariate analysis, logistic regression analysis was performed on selected variables to determine the factors independently associated with BSE practice.

**Results:** The prevalence of BSE was 70.7%. There was no significant association found between socio-demographic, lifestyle or health related factors and BSE. On logistic regression analysis, family history of cancer was not found to be associated with increased practice of BSE (OR 1.02, 95% CI 0.79-1.31). Having attended for mammogram showed a non-significant reduction in the likelihood of doing BSE (Adjusted OR 0.77, 95% CI 0.58-1.02).

**Conclusion:** While we did not identify significant associations with BSE there is a suggestion that attending for mammography reduces women’s interest in BSE. Women need to be advised that BSE and breast awareness alongside routine screening form a two-pronged approach to early detection.

[1] Irish Cancer Society. How to check your breasts 2020 [07 July 2020]. Available from: https://www.cancer.ie/cancer-information-and-support/cancer-types/breast-cancer/how-to-check-your-breasts.

[2] National Health Service. How should I check my breasts? 2018 [07 July 2020]. Available from: https://www.nhs.uk/common-health-questions/womens-health/how-should-i-check-my-breasts/.

## P21 Supporting women with adherence to adjuvant endocrine therapy following breast cancer (SWEET)

### Eila Watson^1^, Mary Wells^2^, Robert Horne^3^, Lucy McGeagh^1^, Jo Brett^1^, Annie Jones^3^, Amy Clarke^3^, Morven Brown^4^, Patricia McCue^4^, Linda Sharp^4^

#### **Correspondence:** Lucy McGeagh

##### ^1^Oxford Brookes University, Oxford, United Kingdom; ^2^Imperial College London, London, United Kingdom; ^3^University College London, London, United Kingdom; ^4^Newcastle University, Newcastle, United Kingdom

**Background**: Adjuvant endocrine therapy (AET) substantially reduces risks of recurrence and mortality in women with ER-positive breast cancer. However, adherence is poor. No effective interventions to support adherence exist. SWEET is developing and evaluating an intervention to improve adherence, improve health-related quality-of-life (HRQoL) and reduce long-term recurrence.

**Method:** SWEET is split into six work streams (WS) spanning six years. WS1 will iteratively develop a person-centred, evidence-based, theoretically informed intervention to support adherence. Including: a tailored consultation with a trained health professional; an app/website including a symptom monitoring tool and other support mechanisms; a three-month follow-up consultation; and regular email/text contact. WS2 will assess the interventions feasibility and acceptability. WS3 will deliver a RCT, with internal pilot and process evaluation. 1018 women at medium/high risk of recurrence will be randomised to usual care or intervention+usual care. Adherence and cancer specific HRQoL will be measured at 6, 12 and 18-months. WS4 will assess intervention cost-effectiveness. WS5will use theory, qualitative research and stakeholder involvement to inform potential NHS scale-up. If WS3 improves adherence, WS6 will assess effectiveness in reducing recurrence at five years. PPI will be integral throughout.

**Results**: Initial work started in May 2020 and is focusing on intervention design and development, using remote approaches to engage patients and health professionals in co-design.

**Conclusion**: This is the first trial of an intervention to improve AET adherence which is powered to detect effects on recurrence. It offers real potential to reduce breast cancer recurrences and deaths thereby benefiting patients, the NHS and society.

## P22 Could psychological and perceptual learning based theories have the potential to transform mammography interpretation programmes

### Helen Yule^1^, Nick Perham^2^, Andrew Watt^2^, Helen Hodgetts^2^

#### **Correspondence:** Helen Yule

##### ^1^Breast Test Wales, Royal Glamorgan Hospital, Cardiff Metropolitan University, Cardiff University, Cardiff, United Kingdom; ^2^Cardiff Metropolitan University, Cardiff, United Kingdom

**Purpose:** The interpretation of a mammogram is a complex visual discrimination task that incorporates both a cognitive and perceptual element to the decision-making process. Current learning strategies involve exhaustive review of thousands of mammograms until levels of expertise are acquired.

Recent advances in learning sciences suggest the potential for improving medical learning and performance. Often neglected, perceptual learning is a fundamental contributor to expertise.  Difficult to teach pattern recognition skills can be systematically accelerated using techniques of perceptual learning. Aside from the “practice makes perfect approach” cognitive science offers a theoretical platform from which to formulate meaningful experiments that could lead to novel training strategies to improve the accuracy and efficiency of training.

**Method:** Learning theorists have demonstrated the benefits of training on easy perceptual discrimination tasks followed by more difficult cases known as the transfer along the continuum. Novice participants will be divided into two groups. Mammograms are digitally displayed according to the level of diagnostic difficulty cascading from easy to hard cases and hard to easy cases. Decisions of normal versus abnormal in a post-test set of 60 mammograms are recorded. Results could show a benefit of training when obvious features are learned first followed by less salient features that require more effort and an extended period of learning.

Further interventions that include re presentation of learning items after a predetermined interval of time or spaced repetition is planned.

**Conclusion:** Acquiring expertise in mammographic interpretation could be accelerated if mammograms are displayed in a specific order.

## P23 Correlation between the shape and size of grade 3 (G3) invasive ductal carcinoma (IDC) in the breast screening population

### Taghreed Toma, Elizabeth Loftus, Youstina Ebrahim

#### **Correspondence:** Taghreed Toma

##### Southend University Hospital NHS Foundation Trust, Southend-on-Sea, United Kingdom

**Aim**: To identify any possible correlation between shape, maximum diameter and patients’ age at time of diagnosis.

Patients and method: Retrospective study of screened ladies over 1-year period including self-referrals. Out of 26012, 202 patients had a confirmed diagnosis of IDC. 43/202 received a treatment for biopsy confirmed G3 IDC. The average age was 62 years. All recalled patients had clinical examination and ultrasound to identify maximum diameter, shape of each lesion and to exclude multi-focality. The shape was classified into rounded, lobulated, ill -defined and calcifications. The diameter of the tumour was divided into 3 groups (<10 mm, 10-30 mm and >30 mm). The patients were divided into 3 groups regarding their age (49-60, 61-70 and >70 years old).

**Results**: The most common presentation was ill-defined (53.48%), followed by rounded (25.58%), lobulated (16.27%) and calcifications (4.65%). We found that 10-30 mm is the most common diameter among all age groups. There was a good correlation between the ultrasound diameter (average 18.7 mm) versus post-surgical pathology diameter (16.2 mm). Most of the patients were in the age group 61-70 (44.18 %).

**Conclusion**: The study demonstrated screen-detected G3 IDC were most commonly ill- defined at time of diagnosis, while the expectations were to find rounded masses as the most common once. The maximum diameter at time of presentation was between 10-30 mm. The highest incidence of G3 cancer was in 61-70 age group.

## P24 To determine the radiological and histological characteristics of biopsy proven DCIS upgraded to invasive cancer after surgery

### Humaira Khan, Kashif Ditta, Yuki Nehikhare

#### **Correspondence:** Humaira Khan

##### Sandwell and West Birmingham NHS Trust, West Bromwich, United Kingdom

**Background:** Biopsy-diagnosed DCIS cases upgraded to invasive malignancy following surgery warrant surgical re-intervention exposing patients to further risk, increase psychological distress and lengthen the treatment pathway. The aim of this audit was to determine our upgrade rate of DCIS and identify radiological and pathological factors associated with this upgrade.

**Method:** Record of all cases of DCIS upgraded to invasive cancer from 2016-2018 were extracted from NHS Breast Screening Programme Central Return Data Set (KC62). Assessment record of all cases was obtained and reviewed for clinical findings, Imaging and histological characteristics. Cases where histology showed suspicion of invasion or micro-invasion on biopsy were excluded.

**Results:** Of the 136 women diagnosed with DCIS on biopsy, 20 cases were upgraded to invasive cancer post- surgery.

75% displayed micro-calcifications ,20% had mass-like morphology, and 5% had architectural distortion. Equal proportions of comedo-necrosis (50%) and cribriform pattern (50%) was seen on biopsy. 90% of invasive malignancies were non-specific type malignancies (NST), followed by mucinous (5%) and mucinous papillary cystic (5%). Majority of cases (50%) were invasive grade 2. No nodal spread and only one case of vascular invasion was recorded. Majority of invasive cancers were ER (85%) and PR (60%) positive, with 15% HER-2 positive.

**Conclusion:** Unfortunately, our results showed no reliable factors to enable us to identify likely cases of invasion amongst DCIS-diagnosed patients. Similar results have been obtained by other studies which failed to identify common characteristics for upgrade ^[1,2,3].^

[1] Hogue JC, Morais L, Provencher L, Desbiens C, Poirier B, Poirier E, Jacob S, Diorio C. Characteristics associated with upgrading to invasiveness after surgery of a DCIS diagnosed using percutaneous biopsy. Anticancer Research. 2014 Mar 1;34(3):1183-91.

[2] Kim J, Han W, Lee JW, You JM, Shin HC, Ahn SK, Moon HG, Cho N, Moon WK, Park IA, Noh DY. Factors associated with upstaging from ductal carcinoma in situ following core needle biopsy to invasive cancer in subsequent surgical excision. The Breast. 2012 Oct 1;21(5):641-5.

[3] Lee CH, Carter D, Philpotts LE, Couce ME, Horvath LJ, Lange RC, Tocino I. Ductal carcinoma in situ diagnosed with stereotactic core needle biopsy: can invasion be predicted? Radiology. 2000 Nov;217(2):466-70.

## P25 Investigation of interval cancer characteristics with comparison to screen detected cancers making inferences concerning prognostic outcome

### Liana Hough, Kathryn Taylor

#### **Correspondence:** Liana Hough

##### Cambridge Breast Unit, Cambridge, United Kingdom

**Purpose**: To investigate characteristics of interval cancers (ICs) and compare them with characteristics of screen detected cancers (SDCs) to add to current evidence and draw inferences on implied prognostic outcomes.

**Methodology:** This was a single centre study, using a non-probability sampling technique to identify the ICs. ICs were age matched with a SDC group and inclusion and exclusion criteria applied. Data on specific characteristics were collected. ICs were sub categorised into true intervals, minimal signs cancers, false negative cancers or occult cancers.

**Results:** There were 106 patients in each group. Forty one percent of ICs presented in the third year after screening. Twenty-nine percent of cancers found in the first year were mammographically occult, 67% of these were lobular carcinomas. Upon further classification of ICs into sub categories the occult group had a larger percentage of dense breast tissue (31%) compared to the true interval group (5%). Thirty two percent of the SDCs were < 10 mm in tumour size compared to 9% of the ICs. Fifty four percent of the ICs were > 20 mm compared to only 12% of the SDCs. We found positive lymph node status in the IC group in 24% of the cases and 6% in the SDC group.

**Conclusion:** The incidence of ICs in the third year after screening suggests that a 2 yearly screening programme in the UK may be beneficial. The IC group had less favourable prognostic features in comparison to the SDC group in line with previous studies.

## P26 Third opinion cancers: Missed or misinterpreted

### Nevine Anandan, Stephanie Walden, Isobel Haigh, Nisha Sharma

#### **Correspondence:** Nevine Anandan

##### Leeds Teaching Hospitals, Leeds, United Kingdom

**Purpose:** Review mammographic features of third opinion cancers and establish learning points to improve sensitivity in detecting subtle cancers in screening population.

**Methods**: Third opinion cancers between 1 Apr 2017 to 31 Mar 2018 with the following data variables recorded:

Mammographic abnormality missed/misinterpreted by first or second reader

Screen round

Technical adequacy of mammogram

Breast density (BIRADS A-D)

Mammographic score of abnormality (R1 Normal - R5 Malignant)

Site and size of abnormality

If abnormality visible on one view only

Correlation with histology

**Results**: 28 third opinion cancers identified.

79 % missed / misinterpreted by the first reader and 21% misinterpreted by the second reader.

82% prevalent round and 18% incident round.

18% considered technically inadequate due to blurring but only two cases felt to be relevant in missing the mammographic abnormality.

7% lesions sited in review areas including overlying the pre -pectoral and the ‘milky way (previously 29% in 2015-16).

25% of lesions were visible on one view only.

In low density breasts, most common feature overlooked was a small mass (≤10mm) accounting for 75% (3/4) of cases.

In extremely dense breasts, most common abnormality missed was calcification accounting for 80% (4/5) of cases.

**Conclusion**: Since the previous audit, we have improved at reviewing 'review' areas.

Be wary whilst dismissing lesions seen on a single view as reasons may include the abnormality lying posteriorly and hence not visible on second view.

Caution while assessing small masses and suspicious calcification on dense breasts even if seen only on a single view.

## P27 Does communication between radiographer and responsible assessor affect the radiographic repeat rate in the assessment clinic?

### Kathryn Taylor

#### Cambridge University Hospitals NHS Foundation Trust, Cambridge, United Kingdom

**Background:** Radiographic practice in the NHS breast screening programme is rigorously monitored. The acceptable standard for repeat rate (RtR) at screening is <3%. Repeat images at assessment are not recorded and the rate unknown. Also, there is little knowledge of communication/decision making between radiographer and responsible assessor (RA) during the assessment process.**Methods:** Questionnaires were developed for stereo/vacuum biopsy assessment cases (S/VBCs) and non-stereo assessment cases (NSCs) where radiographic repeats were taken. The survey covered local radiographic practice including abnormality identification, location, imaging required and associated interactions with the RA. The number of repeats per case was requested including contributory factors. Questionnaires were disseminated to a small number of NHS breast screening units (BSUs) as a pilot.**Results:** Between 5.8.19 and 15.11.19 3424 assessment cases were undertaken in 10 BSUs. For NSCs involving repeats, 90% radiographers identified the abnormality, location and imaging required from documentation on client sheet and/or imaging. In 62% cases there was no discussion with the RA and 74% radiographers decided autonomously to undertake repeats. For S/VBCs 32% radiographers identified the abnormality, location and imaging required from documentation but 58% discussed this with the RA. In 82% S/VBCs it was the RA decision to repeat. RtR was ≤3% in 70% participating BSUs. Sixty percent repeats were NSCs, 40% S/VBCs; difficult abnormality location/client habitus contributed.**Conclusion:** RtR is higher at assessment compared to routine screening relating to a more complex case mix. Consultation with RAs correlated with a lower repeat rate and is recommended for all

## P28 Review of the characteristics of screen detected cancers following discordant recall to improve reader sensitivity with regard to small cancers

### Smitha Krishna Pillai, Humaira Khan, Louise Tromans, Jagdeep Kaur, Edward Goble, Julie Shephard

#### **Correspondence:** Julie Shephard

##### City, Sandwell & Walsall Breast Screening Service, West Bromwich, United Kingdom

**Objectives:** NHSBSP QA standards ^[1]^ state that small cancers should account for 55% of the overall cancers detected. We aim to determine if small cancer detection rate can be improved by reviewing the characteristics of cancers arising from discordant read (where one film reader did not identify the lesion). A secondary outcome will be to develop a case collection of images that demonstrate subtle findings associated with discordant reads for training purposes.

**Methods:** Review of all screen detected discordant cancers from 01.04.2018- 31.03.2020. Each case was reviewed for mammographic abnormality, lesion size, breast density, one/two view presentation, prevalent/incident read, histological subtype, hormone and lymph node status, reader experience and the results recorded.

**Results:** Of 104 screen detected cancers diagnosed from a discordant read, 65(63%) were small cancers. Lesion characteristics were: spiculate/ ill-defined mass (54%), microcalcifications (38%) and other (8%). 70% were invasive,the majority G2 NST. Lymph nodes were involved in 11/104 cases. The majority of discordant cases (70%) were detected by readers with >5 years of experience. 72% of the mammographic abnormalities were demonstrated on both views. 69% were identified in mixed fatty dense breast tissue.

**Conclusions:** Our results highlight the importance of double reading and film reader experience in maximising small cancer detection rate ^[2]^.This has implications for which film readers should be paired and the importance of having experienced film readers undertaking arbitration/ consensus.

[1] Consolidated Standards for NHSBSP QA Standard. April 2017

[2] Cornford EJ, Evans AJ, James JJ, Burell HC, Pinder SE, Wilson ARM. The pathological and radiological features of screen-detected breast cancers diagnosed following arbitration of discordant double reading opinions. Clinical Radiology*.* 2005;60: 1182-1187. Available from: https://doi.10.1016/j.crad.2005.06.003 [Accessed: 12th July 2020].

## P29 What effect does the pre-assessment breast care nurse consultation have on client anxiety levels throughout assessment? A mixed methods analysis

### Paula Hynam

#### Somerset NHS Foundation Trust, Taunton, United Kingdom

Despite NHSBSP^[1]^ guidance, Breast Care Nurse (BCN) resource at assessment may be erratic, service evaluation aimed to demonstrate BCN consult effect on anxiety levels during assessment, reinforcing BCN importance.

**Aims:** Demonstrate anxiety level differences in women, dependent on BCN consult via repeated measures using Spielbergers State Trait Anxiety Index^[2]^ (STAI) questionnaire.

Assess BCN consultation and therefore value to clients via patient questionnaire.

**Methods:** Mixed methods single centre study recruiting 57 women attending assessment clinics over three month period.

State/Trait anxiety levels measured on arrival using full STAI form, followed by repeated measures State anxiety using 6 question short anxiety questionnaire^[3]^. Responses to questions are scored and totalled, differences between scores compared.

Women placed in 2 groups dependant on BCN availability. Group A had pre assessment BCN consult, group B did not. Measurements of anxiety from all women taken on arrival and post assessment. Group A had additional measurement post BCN consult. Comparison of scores between groups undertaken using Wilcoxon signed rank test.

**Results:** Group A - State anxiety significantly decreased post consult compared to baseline, 6.7% (*p* = 0.036).

Both groups - State anxiety significantly reduced post assessment; A-20.7%, (*p* = 0.002), B-17.7% (*p* = 0.008). A demonstrated 3% greater reduction than B.

Biopsy increases state anxiety post assessment, independant of consult.

Group B-60% of women asked wished to have seen a BCN before assessment, highly significant, *p*=0.003.

**Conclusions:** BCN consult significantly reduces anxiety in assessment women, therefore *all* women attending assessment would benefit from BCN consult, as recommended in NHSBSP guidance.

[1] PHE (2016). *NHS Breast Screening Programme. Clinical guidance for breast cancer screening assessment.* NHSBSP publication number 49. Fourth edition. November 2016. London: PHE publications. Pp. 11 – 13.

[2] Spielberger, D., Gorsuch, R. and Lushene, R. (1970) ‘*STAI manual for the state-trait anxiety inventory*.’ Palo Alto, California.: Consulting Psychologists Press.

[3] Marteau, T. and Bekker, H. (1992) ‘The development of a six-item short-form of the state scale of the Spielberger State-Trait Anxiety Inventory (STAI)’, *British Journal of Clinical Psychology*, 31(3), pp. 301-306. doi: 10.1111/j.2044-8260. 1992.tb00997. x.

## P31 Vacuum assisted biopsy/excisions: Do they affect non-operative diagnosis rates?

### Carolyne Geary, Karen Litton, Karen Wilmot

#### **Correspondence:** Carolyne Geary

##### The Great Western Hospitals NHS Foundation Trust, Swindon, United Kingdom

**Purpose:** As per NHSBSP guidelines ^[1]^, departmental protocol is to perform Vacuum Assisted Biopsy/Excision (VAB/E) as a second line biopsy. This is following all B3 stereotactic core biopsies and for other selected cases after MDT discussion. The impact of VAB/E on patient outcome and non-operative diagnosis rates was reviewed over 5 years.

**Method:** All patients undergoing VAB/E during the 5 year period were identified. The 14-gauge core biopsy results and the follow-up 9-gauge VAB/E results were compared. The surgical or non-surgical outcome for these patients was recorded.

**Results:** 113 patients underwent VAB/E: 94 VAB and 19 VAE. Prior to VAB/E 81 had initial B3 core biopsies and 12 B4. Post VAB/E 50 were returned to routine screening. 29 required annual mammographic surveillance, 21 women were upgraded to B5a or B5b and only 4 required diagnostic excisions in theatre. The screening unit’s non-operative diagnosis rate for invasive cancers remained within 1%. However, the non-operative diagnosis rate for non-invasive cancers increased from 79% to 95%.

**Conclusion:** We cannot say how many of these patients would have had a different outcome if the standard 14-gauge stereotactic core biopsy had been repeated. However, all of these patients would have had surgery if no second line biopsy was performed. 70% of patients were reassured with a benign result or increased surveillance. Non-invasive non-operative diagnostic rates have improved since the introduction of VAB/E in this screening unit.

[1] NHS Breast Screening Programme. *Clinical guidance for breast cancer screening assessment* , NHSBSP publication number 49, Fourth edition November 2016. Available at: www.gov.uk/government/publications/breast-screening-clinical-guidelines-for-screening-management [Accessed 29 January 2020]

## P32 Case report - use of VAB to aspirate lactational breast abscess

### Paula Hynam

#### Somerset NHS Foundation Trust, Taunton, United Kingdom

Case report describing management of a large lactational breast abscess during the height of the Covid-19 outbreak utilising Vacuum Assisted Biopsy (VAB) device to aid aspiration, thus avoiding surgical Incision and Drainage.

**Background:** Abscesses are commonly treated with antibiotics and aspiration in an attempt to avoid surgical intervention if possible. The purpose of treatment is the removal of infection and pus as rapidly as possible to avoid further complications [1]. This case report describes the process of VAB aspiration of a persistent large lactational breast abscess. Surgical colleagues were unwilling to take the patient to theatre due to Covid-19.

**Presentation:** A breast feeding woman in her twenties, 15 weeks post-partum was admitted with left UOQ breast lump and accompanying pink skin with a swollen warm firm breast consistent with lactational abscess [2]. Patient was systemically unwell, spiking temperature, unable to feed/express from symptomatic breast. Administered IV antibiotics (flucloxacillin) with analgesia. US demonstrated a 100x60mm collection, repeated aspirations had variable success with needle blockages problematic. To avoid surgery, VAB aspiration under US guidance was performed.

**Conclusion:** VAB successfully aspirated over 200ml of material from the abscess which facilitated patient recovery. This was the first time our institution had utilised VAB in this manner. Literature search found 2 studies that utilised VAB in this way, both concluding VAB to be a safe and viable alternative to surgical intervention in the case of larger (over 5cm) abscesses [3].

[1]. Kang, Y and Kim, Y (2015) ‘Comparison of needle aspiration and vacuum-assisted biopsy in the ultrasound-guided drainage of lactational breast abscesses’ *Ultrasonography.*2016;35:148-152

[2]. Mahoney, M (2014) ‘Breast Emergencies: Types, Imaging Features, and Management.’ *American Journal of Roentgenology.* 4, 202. *pp* W390-W399.

[3]. Chen, C, Luo, L, Gao, D, Qu, R, Guo, Y, Huo, J and Su, Y. (2019) Surgical drainage of lactational breast abscess with ultrasound‐guided Encore vacuum‐assisted breast biopsy system.*The Breast Journal,* 2019 Sep-Oct; 25(5): 889–897.

## P33 An audit of 76 ultrasound guided (10g) vacuum-assisted biopsies (VAB)

### Sue Garnett

#### University Hospital Coventry, Coventry, United Kingdom

This new procedure has been audited to assess outcomes, highlight complications and guide safe expansion to vacuum excision as a service improvement.

Statistics were collected, including lesion size, distance from nipple and skin, number of cores, biopsy results, final histology and outcomes.

Results of the 70/76 cases that were second line biopsies have been analysed.

16% (n=11) of cases were fibroadenomata, benign breast change or sclerosing lesions.

30% (n=21) were papillomata with no atypia and avoiding surgery.

27% (n= 19) were upgraded to cancer pre-surgically.

27% (n=19) were other non malignant lesions of which 5 cases were ADH or papilloma with atypia.

11% had less than 6 cores, done in the early years or were halted procedures; 68% had 7-17 cores, and 21% had 18+ cores, now done as the routine minimum.

Five complications were minor only of pain and haematoma; only one of these was 0.5cm from the nipple and 0.8cm from the skin.

Sizes ranged from 3-80mm

65% were well defined masses.

**Conclusion**: 25% of cases became cancer ensuring therapeutic surgery and 67% cases were discharged avoiding excisional surgery.

Safe distance from the nipple and skin are important considerations for this procedure.

Ultrasound VAB procedures are now routinely undertaken, for lesions easily identified, reducing the need for stereotactic approach. VAE is slowly evolving, protocols in place including moving to 7g. The poster will illustrate the safety and accuracy of this efficient procedure.

## P34 Audit of whole breast ultrasound in patients with breast cancer in a symptomatic unit

### Tracy Durkin, Nerys Forester

#### **Correspondence:** Tracy Durkin

##### Calderdale Hospitals NHS Trust, Halifax, United Kingdom

Historically, we have performed bilateral whole breast ultrasound (WBUS) at the time of breast cancer diagnosis. Prior to changing practice to comply with RCR guidelines we audited the role of WBUS in our unit.

**Methods:** Review of all imaging findings following all cancers diagnosed in 2018.

**Results:** 244 patients were identified with unifocal carcinoma in 2018. 192/244 patients had WBUS performed at the time of diagnosis, in 166 no further lesions were identified.

26/192 patients had an abnormality seen on the contralateral WBUS. 15/26 were cysts. In 11/26 patients a biopsy was performed which identified 6 FAs and 5 extra foci of cancer. In 9/11 biopsies, the lesion could be identified on the initial mammogram. 2 lesions identified solely on WBUS were benign following biopsy.

Despite WBUS, 16 patients still required a 2nd look US following MRI scan. In 10/16 patients this was of the contralateral breast. All lesions were identified and biopsied and malignant in 4/10.

7/16 patients required a 2nd look of the ipsilateral breast, of which 5/7 were malignant on biopsy. One of these patients had multifocal cancer diagnosed despite an initial normal WBUS.

**Conclusion**: 14 extra foci of cancer were identified in 249 patients with symptomatic breast cancer. These were all identified by mammography or MRI. WBUS did not identify any additional malignant lesions not seen on other modalities. WBUS does not add any diagnostic value for multifocal breast cancer diagnosis; it can be falsely reassuring and increase unnecessary biopsies.

## P35 2nd look ultrasound after breast MRI in pre-operative patients

### Becky Roberts

#### The Rotherham NHS Foundation Trust, Rotherham, United Kingdom

**Background:** Magnetic resonance imaging (MRI) is known to be the most sensitive imaging modality for examining the breast. Due to it's low specificity breast MRI is often used as a problem solving tool, to examine those at high risk of developing breast cancer or to monitor those on neo-adjuvant treatment regimes. Targeted breast ultrasound is the imaging modality of choice in this centre to further evaluate additional lesions detected on breast MRI. Although playing an essential role in the development of a treatment plan- these additional imaging stages inevitably slow down the diagnostic process, and there is a feeling that referrals for breast MRI may be increasing unnecessarily.

An audit was performed to establish the frequency and outcome of 2nd look ultrasound following breast MRI in those with an already diagnosed breast malignancy, as well as the clinical indication for breast MRI.

**Methods:** A retrospective audit was performed of all breast MRI performed in this centre within a 2 year period on women with at least 1 biopsy proven malignancy. The referral type was plotted against those recommended by the European Society of Breast Imaging (ESOBI) ^[1].^ Those exams requiring 2nd look ultrasound were reviewed with ultrasound results and additional biopsy rates noted.

**Results:** 87 breast MRI were reviewed from the 2 year audit period. 19 were referred for 2nd look ultrasound, with 12 requiring additional biopsy. 4 new malignancies were identified. No women were referred for MRI biopsy.

Referrals for breast MRI were in line with those from ESOBI guidance.

[1] Mann R, Kuhl C, Kinkel K, Boetes C. Breast MRI: guidelines from the European Society of Breast Imaging. European Radiology. 2008; 18(7):1307-1318

## P37 Is the 90° lateral projection still useful in diagnostic mammography when Digital Breast Tomosynthesis (DBT) is available?

### Ciara Dowling^1^, Dylan Wynn-Jones^1^, Anthony Dennis^2^

#### **Correspondence:** Ciara Dowling

##### ^1^Breast Test Wales, Cardiff, United Kingdom; ^2^Kingston University, London, United Kingdom

**Background:** Mammography is the gold standard for investigation of breast abnormalities but is limited by overlapping tissue obscuring real lesions or mimicking malignancy ^[1,2].^ The traditional 90° lateral projection and Digital Breast Tomosynthesis (DBT) provide similar information when investigating possible lesions identified on standard mammography. This study aims to investigate the frequency of use and usefulness of the 90° lateral projection in breast screening assessment clinics where DBT is available.

**Methods:** A self-administered questionnaire was designed and piloted. Clinicians within a screening organisation were invited to complete one questionnaire per case in assessment clinics during a 6-week period.

**Results:** Twelve clinicians from two of the three invited regions participated. 231 questionnaires were included in the dataset. Lateral projection and DBT were used frequently, in 81.8% (n=189) and 83.5% (n=193) of cases respectively. They were used to complement each other as evidenced by varied indications. Lateral projections and DBT were reported ‘very useful’ or ‘useful’ in most cases, 65% and 79.3% respectively.

**Conclusions:** The lateral projection remains frequently used when DBT is available. Radiation dose of performing both is justified by varied indications for use and both were reported useful in most cases. Increased use of the lateral DBT projection could combine the benefits of an orthogonal projection to indicate lesion location and those of DBT in characterising lesions.

[1]. Roth, R. et al. Digital Breast Tomosynthesis: Lessons Learned from Early Clinical Implementation. Radiographics [Internet]. 2014. [cited 11 February 2019]; 34(4), p.E89-E102. Available from: https://pubs.rsna.org/doi/full/10.1148/rg.344130087

[2]. Giess, C., Frost, E. and Birdwell, R. Interpreting One-View Mammographic Findings: Minimizing Callbacks While Maximizing Cancer Detection. RadioGraphics [Internet]. 2014. [cited 11 February 2019]; 34(4),928-40. Available from:https://pubs.rsna.org/doi/10.1148/rg.344130066

## P38 Imaging theatre specimens: The impact of imaging methods on surgical re-excision rates

### Carolyne Geary, Karen Litton, Frances Vincent, Ruth Fry

#### **Correspondence:** Carolyne Geary

##### The Great Western Hospitals NHS Foundation Trust, Swindon, United Kingdom

**Purpose:** In 2018 digital breast tomosynthesis (DBT) equipment was installed in the department. Published literature claimed DBT a superior imaging technique for perioperative specimen imaging ^[1]^. A combination of DBT and full field digital mammography (FFDM) was introduced and audited.

**Method:** All surgical specimens in a 9 month period were imaged using FFDM/DBT or in theatre using a specimen x-ray cabinet (SXC). Cases were separated into 3 month groups, before, during and after equipment changeover. All FFDM/DBT images were reviewed by the radiology team and verbally reported to theatre. Data regarding imaging method and specimen margin status was collected. Results were discussed within the multidisciplinary team and a further 3 month period of data collection was agreed upon.

**Results:** During the initial 9-month audit 236 specimens were imaged. 40 needed surgical re-excision. 183 specimens were imaged using FFDM/DBT and 53 using SXC. The SXC images showed a higher percentage of re-excisions (26.42% versus 14.2% in FFDM/DBT). Post introduction of DBT (88 specimens) the re-excision rate for FFDM/DBT fell from 15.9% to 12.7%, suggesting DBT may be beneficial, Post MDT discussion showed a reduction of specimens imaged using SXC, (21.7% to 13.6%), due to imaging method selection by surgeons.

**Conclusion:** There has been a reduction in re-excision rates since the introduction of DBT. Due to significant differences in re-excision rates between FFDM/DBT versus SXC, auditing has prompted a change of surgical practice and imaging protocols. However, non-calcifying DCIS could be a confounding variable to this

[1] Amer, H.A., Schmitzberger, F., Ingold-Heppner, B., Kussmaul, J., El Tohamy, M.F., Tantawy, H.I., Hamm, B., Makowski, M. and Fallenberg, E.M. ‘Digital breast tomosynthesis versus full-field digital mammography—Which modality provides more accurate prediction of margin status in specimen radiography?’, European Journal of Radiology, (2017) Volume(93), pp.258–264. Available at: DOI:10.1016/J.EJRAD.2017.05.041 [Accessed: 31 January 2020].

## P40 A service evaluation of the accuracy of axillary ultrasound and MRI in determining lymph node metastasis in patients with breast cancer

### Faisal Majid^1^, Judy Davis^1^, Andrew England^2^

#### **Correspondence:** Judy Davis

##### ^1^Sandwell and West Birmingham NHS Trust, West Bromwich, United Kingdom; ^2^Keele University, Keele, United Kingdom

**Purpose**: Axillary lymph node metastasis is seen as a key prognostic factor for breast cancer patients. [1] Pre-operative diagnosis of axillary lymph node metastasis can ensure patients receive the appropriate axillary surgery and can prevent the need for further surgery. [2] This study assessed the accuracy of ultrasound, MRI and ultrasound guided core biopsy in diagnosing axillary lymph node metastasis pre-operatively. The results will aim to refine our current clinical practice.

**Methods**: Ultrasound and MRI data was retrospectively analysed from breast cancer cases diagnosed between January 2017 and December 2019. The results were correlated to the final histological outcomes from the surgery.

**Results**: Two hundred and fifty eight cases were included in the study, 107 (41.5%) had evidence of lymph node metastasis on final histology. Ultrasound was compared to MRI to establish which imaging modality was most accurate at detecting lymph node metastasis. Ultrasound was demonstrated to have a sensitivity of 67%, specificity of 87%, PPV 79%, NPV 79% and an Accuracy of 79%. MRI was demonstrated to have a sensitivity of 76%, specificity of 84%, PPV 77%, NPV 83% and an Accuracy of 80.6%. 103 of the cases had ultrasound guided core biopsy of an abnormal node to establish lymph node metastasis. The overall sensitivity of lymph node core biopsies was 86%, specificity was 100%, PPV 100%, NPV 68% and accuracy 89.3%.

**Conclusion**: There was no statistical difference between the performance of ultrasound to MRI in the detection of lymph node metastasis. No change in clinical practice.

[1]     Valente SA, Levine GM, Silverstein MJ, Rayhanabad JA, Weng-Grumley JG, Ji L, Holmes DR, Sposto R, Sener SF. Accuracy of predicting axillary lymph node positivity by physical examination, mammography, ultrasonography, and magnetic resonance imaging. Annals of surgical oncology. 2012 Jun 1;19(6):1825-30.

[2] NHS Breast Screening Programme, Clinical guidance for breast cancer screening assessment. NHSBSP publication number 49, Fourth edition November 2016

## P41 Audit of breast skin abnormalities referred to the one stop clinic: Is imaging contributory?

### Sylvie Flais, Gita Ralleigh, Neelofer Zaman, Angela Gupta

#### **Correspondence:** Sylvie Flais

##### Charing Cross Hospital, London, United Kingdom

**Purpose:** Patients presenting to the breast clinic with clinically assessed skin lesions are frequently referred for additional diagnostic US scan. 8 months of radiology activity were audited to assess whether US contributed to the management of these lesions.

**Methods:** The reports of US performed in the rapid diagnostic clinic between 1/01/2020 and 31/08/2020 were reviewed retrospectively. A case was included in the audit if the clinical details or the imaging details suggested that the presenting symptom was due to a skin lesion. Patients who presented with a malignant mass involving the skin were excluded.

**Results:** 167 cases were identified out of 4442 patients referred to the breast rapid diagnostic clinic in our institution. Most cases were clinically assessed as due to benign causes. In the large majority of cases there was no further action following US imaging. There was one adverse event: a lesion thought to be an epidermoid cyst at presentation was later proven to be an invasive carcinoma (increasing size). Another skin lesion that appeared very vascular on US in a patient who had previously been treated with radiotherapy was biopsied and proved to be an angiosarcoma.

**Discussion:** US features of skin lesions are reviewed. We discuss the contribution of US and its influence on further management.

## P42 Axillary ultrasound accuracy in identifying metastatic nodes in patients with breast cancer

### Monica Patil, Nikhil Patel, Clare Peacock, Shalini Wijesuriya, Rema Wasan, Rumana Rahim, Bhavna Batohi, Michael Michell, Juliet Morel, Keshtra Satchithananda

#### **Correspondence:** Monica Patil

##### King's College Hospital, London, United Kingdom

**Background**: Axillary ultrasound (AUS) sensitivity in detecting axillary lymph node (ALN) metastases is low with no standard cortical thickness threshold to indicate nodal sampling.^[1,2,3,4]^ The RCR suggests a sensitivity of 50% for AUS in detecting ALN metastases. Nodal disease confirmed preoperatively results in a 'fast-track' to axillary node clearance (ANC).

Following a local departmental audit in 2017, we changed our cortical thickness threshold from 2mm to 3mm based on a predicted increase in specificity with only a modest drop in sensitivity.

**Methods**: Retrospective analysis of 100 screen detected and symptomatic breast cancers in 2019 where final axillary staging with sentinel lymph node biopsy or ANC was available. Findings were correlated with AUS and core biopsy results. Those receiving neoadjuvant chemotherapy were excluded.

**Results**: The change in the cortical thickness threshold reduced the sensitivity from 65.7% to 60%, which is still well above the expected RCR standard of 50%. The specificity and accuracy improved from 83.9% to 95.7% and 78.1% to 85% respectively. There were 12 false negative AUS cases: 8 were invasive lobular carcinoma (ILC), of which 5 had low burden disease (1-2 nodes involved), and 4 were invasive ductal carcinoma, all of which had low burden disease.

**Conclusion**: The recommendation is to maintain a cortical thickness threshold of 3 mm, quantify disease burden on AUS and accept a slightly lower sensitivity but higher specificity. Further work needed to investigate whether a ‘second look’ AUS following breast MRI for ILC is of any value in detecting ALN metastases.

[1] Balasubramanian I, Fleming C, Corrigan M, Redmond H, Kerin M, Lowery A. The diagnostic accuracy of ultrasound guided fine needle aspiration and core needle biopsy in diagnosing axillary lymph node metastasis in the post Z011 era: A systematic review and meta-analysis. International Journal of Surgery. 2018;55:S18.

[2] Ganott M, Zuley M, Abrams G, Lu A, Kelly A, Sumkin J et al. Ultrasound Guided Core Biopsy versus Fine Needle Aspiration for Evaluation of Axillary Lymphadenopathy in Patients with Breast Cancer. ISRN Oncology. 2014;2014:1-9.

[3] Ahmed M, Jozsa F, Baker R, Rubio I, Benson J, Douek M. Erratum to: Meta-analysis of tumour burden in pre-operative axillary ultrasound positive and negative breast cancer patients. Breast Cancer Research and Treatment. 2017;166(2):337-337.

[4] Fernandez B, Paish E, Green A, Lee A, Macmillan R, Ellis I et al. Lymph-node metastases in invasive lobular carcinoma are different from those in ductal carcinoma of the breast. Journal of Clinical Pathology. 2011;64(11):995-1000

## P43 Mobile specimen radiography- is image quality sufficient for 'point-of-care' use without specimen radiographs by conventional digital mammography?

### Charul Patel, Hannah Constanti, Patricia Marin-Crespo, Helen Richardson, Adelola Oseni, Anita Rhodes

#### **Correspondence:** Charul Patel

##### Kingston Hospital NHS Foundation Trust, London, United Kingdom

**Introduction**: The use of mobile digital specimen radiography systems (DSR) is advocated following wire localisation of impalpable lesions, to confirm total target excision and reduce re-excision rates ^[1].^

**Aim:** Compare image quality and accuracy for depicting various targets (mass, distortion, microcalcification, marker clip) with DSR (Faxitron) to conventional digital mammography (CDM, GE) using compression and magnification, as the reference standard.Assess DSR and CDM specimen radiographs in predicting complete excision with histological correlation as the reference standard.

**Method**: Retrospective audit of specimen radiographs taken between August 2018 - January 2020 with DSR and CDM, scored by 3 teams (breast radiologists and experienced film reader) for: image quality, target visibility and complete excision, on a 3-point scale.

**Results**

**Cohort:** 56 specimen radiographs depicting 63 targets.

**Image quality**: scored as good or moderate of 85% DSR vs 95% CMD radiographs.

**Target Visibility:** 90% of all targets were ‘present’ or ‘probably present’ with both modalities. 10% were not seen with either. 14% were mammographically occult (US visible only or marked by clip, post neo-adjuvant treatment). Some readers scored clips as targets.

**Complete Excision:** Margins were scored as positive on 27.5% of DSR and 23% of CDM radiographs (average for all readers taken).

22% of margins were confirmed positive on specimen histology ^[2].^

**Conclusion**: Excellent target visibility and comparable margin assessment is achievable with DSR and CDM.

Experience and use of image optimization tools can improve confidence in DSR interpretation.

DSR, based in theatre, can provide 'point-of-care' assessment of excision specimens.

[1] Sibbering M, Watkins R, Winstanley J, Patnick J. Quality assurance Guidelines for surgeons in breast cancer screening. NHSBSP Publication no 20, 4th edition; March 2009 https://assets.publishing.service.gov.uk/government/uploads/system/uploads/attachment_data/file/465694/nhsbsp20.pdf

[2] Jeevan R, Cromwell DA, Trivella M, et al. Reoperation rates after breast conservation surgery for breast cancer among women in England: retrospective study of hospital episode statistics. BMJ 2012;345:e4505 https://www.bmj.com/content/345/bmj.e4505

## P44 Second reporting of symptomatic mammograms in one stop breast clinic

### Asha Ramakrishnan, Leanne Greenwood

#### **Correspondence:** Leanne Greenwood

##### Mid Yorkshire Hospitals Trust, Wakefield, United Kingdom

**Background**: One stop breast clinics are a high pressure, busy environment, where the likelihood of missing an abnormality is high. Current practice at our DGH one stop clinic is that imaging is reported by a single consultant radiologist or radiographer. The aim of this audit was to identify whether there would be a benefit to second reporting of symptomatic clinic mammograms.

**Methods**: All one stop clinic mammograms during a 6 month period were second reviewed to see if there was any discordance with the original report. If a discrepancy was identified, a third review was performed. If the second and third review agreed there was a discrepancy, further details were obtained regarding further investigations, follow up and biopsy results.

**Results**: 1452 patients underwent mammography in the 6 month time period. 13 discrepancies were identified between the original mammogram report and second/third review. Of these, 4 have not currently been followed up, 2 patients have died, and 7 have undergone further follow up and biopsy. Of the 7 who have had biopsies, 4 of these were found to have invasive cancer, 1 a diagnosis of DCIS and 2 had benign disease. 3 patients had a delay in the diagnosis of DCIS or invasive cancer (range 3 months – 14 months delay).

**Conclusions**: Our review suggests double reporting of imaging in symptomatic clinics could reduce error and increase sensitivity. It may lead to a reduction in missed cancers and earlier detection of incidental cancers, a benefit to both patients and the trust.

## P45 Evaluation of the use of targeted breast ultrasound in women aged 24-29 with clinically normal (P1) breast tissue attending the one stop breast clinic between October 2018 -- April 2019

### Imelda Marshall^1^, Shazia Khan^2^, Paul Burrows^2^, Lisa Brown^2^, Elizabeth Cooke^2^

#### **Correspondence:** Imelda Marshall

##### ^1^Bradford Teaching Hospitals NHS Foundation Trust, Bradford, United Kingdom and University of Leeds, Leeds, United Kingdom; ^2^Bradford Teaching Hospitals NHS Foundation Trust, Bradford, United Kingdom

**Background:** Willet et al. (2010) recommends ultrasound as the primary imaging tool in women aged <40, where a triple assessment approach should be adopted where deemed necessary^[^¹^]^. RCR (2019) states a scale of U1-5 should be adopted to indicate level of concern, U1 representing normal breast tissue and U5 showing a high level of suspicion^[^²^]^. With clinical demand increasing into the breast clinic, can imaging referrals be further streamlined in this younger cohort of patients where a normal ultrasound report may be associated with a normal clinical breast exam.

**Aims**

- To identify the ultrasound classification associated with P1 examinations.
- Were these P1 cases true representation of a normal clinical examination.

**Methods:** The Computerised Radiology Information System (CRIS) system generated a list of women who had attended clinic between October 2018- April 2019 aged between 24-29 who were referred for an ultrasound and assigned a clinical grade of P1.

**Results:** 140 women met the inclusion criteria. Of these 85/140 (61%) were classified as P1. These were further subdivided into their ultrasound classification; Normal (U1) was 74/85 (87%) Benign (U2) 8/85 (9%) and finally 3/85 (4%) (U3) strictly indeterminate. However, 11 cases were not strictly P1, most included lump and skin thickening. Of the true P1 patients 100% had a U1 ultrasound.

**Conclusion:** Where a clinical indication of P1 was assigned this was followed by a normal ultrasound classification of U1 in 87% of cases. Care should be taken in this age group to ensure concordance of the clinical level of suspicion with the ultrasound classification.

[1] Willet et al. (2010) Best practice diagnostic guidelines for patients presenting with breast symptoms. [online] Accessed 27th January 2020. Available from URL: https://associationofbreastsurgery.org.uk/media/1416/best-practice-diagnostic-guidelines-for-patients-presenting-with-breast-symptoms.pdf

[2] RCR (2019) Guidance on screening and symptomatic breast imaging- Fourth edition. [online] Accessed 27th January 2020. Available from URL: https://www.rcr.ac.uk/system/files/publication/field_publication_files/bfcr199-guidance-on-screening-and-symptomatic-breast-imaging.pdf

## P46 Is diagnostic imaging of the breast possible with a Magseed in situ?

### Jennifer A Summersgill, Jennifer A Macfarlane, Mark Worrall, Andrew Evans, Sandra Gawley, Yee Ting Sim

#### **Correspondence:** Jennifer A Summersgill

##### NHS Tayside, Dundee, United Kingdom

**Purpose:** To quantify the CT and MRI susceptibility artefact of a 5mm Magseed breast marker constructed of surgical grade stainless steel^[1]^. This will determine whether a patient with this marker in situ can undergo diagnostic breast imaging.

**Methods:** Phantom testing was done on MRI^[2]^ and CT^[3]^. Additionally a healthy volunteer was scanned using MRI with the marker on the skin surface. All MRI was performed at 3T using our clinical breast protocol which included spin echo, gradient echo, DWI and dynamic sequences. CT scanning was performed with filtered back projection and iterative reconstruction to compare techniques.

**Results:** On MRI spin-echo sequences the artefact diameter was 4.1cm in-vivo, consistent with the manufacturer guidance^1^. For gradient echo and DWI the artefact ranged from 4.7 cm to 5.8 cm. Fat saturation was unsuccessful with the marker in dynamic imaging resulting in a much larger artefact of 9.5 cm. 3D surface rendering of the in-vivo MRI showed a butterfly distribution of the artefact. On CT the artefact was limited to the size of the marker and remained unaffected by reconstruction technique.

**Conclusion:** Non-breast MRI with the marker in situ can be performed safely while retaining diagnostic quality of images. The presence of the marker in breast MRI causes significant artefact up to 9.5cm from the marker rendering anatomy within this region non-diagnostic. Patients with this marker in situ are unlikely to benefit from an MRI breast exam until after the marker has been removed. CT imaging is unaffected by the presence of the marker.

**Fig. 1:**
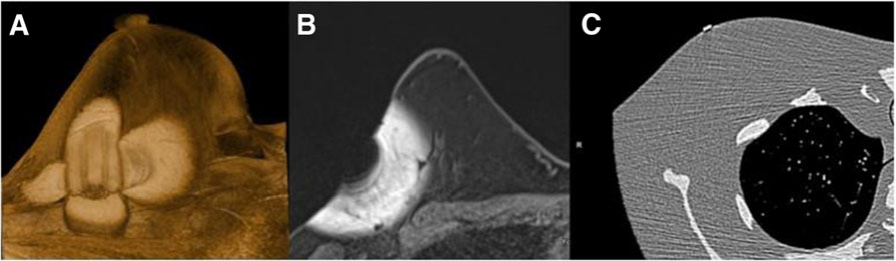
**(abstract P46). See text for description.**

[1]. **Endomagnetics Ltd, Cambridge.** Magseed for use with Sentimag. Instructions for Use, Page 1.

[2]. **Inc., Supertech.** ACR MRI Phantom - ACR-PH1 - ACR-PHE. *supertechx-ray.* [Online] Supertech, Inc. [Cited: 15/10/2020] https://www.supertechx-ray.com/MRI/MRI-ACR-PH-1-E.php.

[3]. **Rothband, Lancashire.** Kyoto Multipurpose Chest 'Lungman' Phantom. [Online] Rothband [Cited: 15/10/2020] https://www.rothband.com/product/kyoto-lungman-phantom/

## P47 Magseed localisation for non palpable breast lesions: Trial experience

### Becky Roberts, Holly Scotson

#### **Correspondence:** Becky Roberts

##### The Rotherham Foundation Trust, Rotherham, United Kingdom

**Background:** Breast cancer screening programmes have led to an increase in the detection of non-palpable breast lesions which traditionally have been localised with wire guidance, placed under ultrasound or stereo taxis on the day of surgery. This relies on close liaison with surgical and radiology teams, which with ever increasing demands on these teams can prove difficult. These difficulties have led to cancelled operations and service delays. Furthermore complications with wires such as the potential for them to move once placed within the breast may lead to incomplete excision.

The Magseed is a relatively new localisation technique that can be placed up to 30 days prior to surgery, and has the potential to increase radiology and surgical flexibillity and thus improve the service offered to our patients.

**Method:** The Magseed was trialled within our NHS Trust for a short period in 2019 and here we present an audit of the trial results, with a case study pictorial review.

**Conclusion:** The Magseed localisation device allowed for easy and accurate placement in radiology, with complete surgical margins in all post operative specimens.

The increased flexibility with placement time will allow for improved access for localisation and surgery.

## P48 Would it be safe to avoid tissue sampling in ultrasound benign breast lesions (U2) in women <30 years old?

### Georgiana Zamfir^1^, Ketki Khadtare^1^, Kirsten Stafford^2^, Emily Daulton^2^, Fiona Hearn^2^, Philippa Skippage^2^

#### **Correspondence:** Georgiana Zamfir

##### ^1^Ashford and St Peter's NHS Foundation Trust, Chertsey, United Kingdom; ^2^Frimley Park Hospital, Frimley, United Kingdom

**Background:** Histopathology is the gold standard for diagnosis of breast masses, but it is an invasive procedure and places considerable stress on a system with limited resources. Best practice diagnostic guidelines for symptomatic breast imaging, widely followed in the NHS, state that benign lesions in women <25 years old do not warrant needling. We carried out this study to determine if this age limit can be safely increased to 30 years. Accurate ultrasound grading is crucial for this; therefore, we also studied grading variability between ultrasound operators within the breast imaging department.

**Methods:** We correlated ultrasound grading and pathological reports for tissue sampling performed on all women <30 years old in our Breast Unit over a year period. Also, a blinded lesion characterisation assessment was completed by breast radiologists who regularly participate in breast ultrasound, to establish if the Stavros criteria applied were accurate.

**Results:** 81 ultrasound-guided samplings (48 core biopsies and 33 fine needle aspirations (FNAs) were performed for women <30 years old (39 U2 and 41 U3 lesions). None of the U2 lesions were malignant on pathology. 11/ 33 FNAs were non-diagnostic and patients were recalled for a biopsy, all of which proved benign. 39/ 41 U3 lesions were benign on pathology.​

**Conclusion:** Tissue sampling can be safely avoided for U2 lesions in women less than 30 years old, provided strict ultrasound criteria are adhered to and hence, we propose that the current national age limit of 25 years be reviewed and consideration be given to it being increased to 30 years.

## P49 Examples of the great mimickers of breast carcinoma and their sonographic appearances

### Samantha West

#### East Lancashire Breast Screening Unit, East Lancashire Hospitals NHS Trust, Burnley, United Kingdom

Breast carcinomas can take on a multitude of appearances that display the features of a true carcinoma. These being: shape, orientation, echo pattern and posterior features.

However, some benign entities can mimic these appearances on ultrasound.

This pictorial review demonstrates some of the great mimickers that display the worrying features.

Diabetic Mastopthy - Disease with fibro inflammatory processes of the breast. Features can include hard, irregular hypoechoic mass with posterior shadowing.

Fat Necrosis - Known as a benign non-supparitive inflammatory process. This process occurs due to breast trauma. Some of the common causes are: radiotherapy, surgery or trauma. Sonographically, the appearance may display an irregular complex mass, edge shadowing or a hyper-echoic irregular mass.

Tuberculosis - The most frequent mode of infection is spread from the axillary nodes. Appearances can present as nodular, diffuse and sclerosing. The nodular type of Tuberculosis can manifest as an ill defined hypoechoic mass. The diffuse type can simulate inflammatory carcinoma, and the sclerosing type can be associated with areas of architectural distortion.

Granular Cell tumour - A benign neoplasm derived from perineural scwann cell of peripheral nerves. On ultrasound, the appearances can present as an irregular or ill defined mass with posterior acoustic shadowing.

Fibromatosis - This is a benign tumour that can occur in the breast. The definitive eitology is unclear but can be associated with Gardeners' syndrome. On Ultrasound, this can present as an irregular hypoechoic mass with a thick echogenic rim and posterior shadowing.

[1] Kim, Y.R., H.S. and Kim, H.W., 2015. Are irregular hypoechoic breast masses on ultraouns always malignancies ? : a pictorial esaay. Korean journal of radiology, 16 (6), pp.1266-1275.

[2] Kopans,D.B.,2007. Breast Imaging. Lippincott Williams and Wilkins.

[3] Multiparametric Ultrasound Diagnosis of Breast Diseases. Gennady.T.Sukhikh, edited by Alexander.N.Sencha. Springer International Publishing AG. 2018

[4] https://www.google.com/url?sa=i&source=images7cd=&cad-rja&uact=8&ved=2ahUKEwjT7KKuK3kahVM-YUKHbRSAuQQjhx6BagBEAI&url=https%3A%2F%2Fradiopeadia.org%2Fcases%2Fdiabeticmastopthy&psig=AOvVaw1kR2n_8EbOveFIjmJaAr9H&ust=1567352234839005 (accessed Sept 2019)

[5] Jagannathan, D.M., 2015. Benign granular cell timour of the breast: Case report an literature revies. Radiology case reports, 10 (2), p.116.

[6] https://www.google.com/url? sa=i&source=images&cd=&cad=rja&uact=8&ved=2ahUKEwjr_IHpwk3kAhUqxoUKHQ75BIMQjkxBAgBEAI&url=https%3A%2F%2Fradiopaedia.org%2Farticles%2Fbreast-abscess&psig=AOvVawONt3jmiTiiS6-9RmZQ2Nle&ust=1567354533570738 (accessed Sept 2019)

[7] Prapruttam, D., Hedgire,S.S., Mani,S.E. Chandramohan, A., Shyam kumar, N.K. and Harisinghani, M., 2014, June. Tuberculosis - the greast mimicker. In seminars in ultrasound, CT and MRI (volume number 35, No. 3, p. 195-214). WB Saunders

[8] Image Source : hhttp://radiopauedia.org/cases/fat-necrosis-breast-4 (accessed Sept 2019)

[9] Khaled Abdelwaheb, Omar Hamdy, Mona Zaky, Nirmeeen Megahed, Saleh Elbalka, Mohammed Elmetwally and Adel Denewer, 2017. Breast Fibromatosis, an unusual Breast Disease.

## P50 Innovative ways of training delivery in post COVID era

### Soujanya Gadde

#### National Breast Imaging Academy, Manchester, United Kingdom

Teaching and training are key components of everyday work for most of the breast imagers in order to provide a high quality and well trained work force for the future. They are also often the most rewarding aspects of one’s career. Unfortunately, COVID 19 has put an unexpected spin on how training has been delivered over the past few decades. Need to limit human contact meant all ‘face to face’ and ‘being on the shop floor’ sessions had to come to an abrupt stop. As the pandemic hit, attention was focused predominantly on essential service delivery whilst training had to be put on hold for a brief time. However, most trainers quickly realised the detrimental effect this was having on an entire generation of work force and started to look for alternative options. A new world of virtual platforms and virtual interaction has started becoming the new norm for delivery of training.

The aim of this poster is to highlight various freely available online platforms and wed based resources that can help in to deliver training in a safe, effective and time efficient manner. We share our experience in using the more popular platforms with an emphasis on the pros and cons to help the reader choose the most appropriate ones for their purpose. We also share alternative ideas on how to effectively deliver training in a clinical setting during COVID restrictions.

## P51 Challenges of polyacrylamide gel (PAAG) breast augmentation

### Joleen Kirsty Eden, Suzanne Gawne, Richard Dobrashian

#### **Correspondence:** Joleen Kirsty Eden

##### East Lancashire Hospitals NHS Trust, Burnley, United Kingdom

The injectable breast filler Polyacrylamide hydrogel (PAAG) was widely used in China since the 1980s with as many as 300,000 women subjected for cosmesis and reconstruction following cancer^.[1]^ The procedure requires no anaesthesia, often injected by non-medical professionals.

No safety clinical trials were conducted and in 2006 the Chinese State Food and Drug Administration prohibited the clinical application following significant evidence of neurotoxic and teratogenic monomers residual in the synthesis of PAAG^.[2]^

Although now withdrawn, many patients are developing on-going associated complications of PAAG and presenting worldwide to surgeons unfamiliar with the treatment, necessitating complex surgery. Management of PAAG is not standardised and often directed by the radiological appearances.

A case study is reported discussing the associated challenges of PAAG with recommendations gathered from the literature.

Radiological imaging can mimic malignancy with inflammatory appearances, whilst simulating silicone implants and the features of Breast Implant Associated – Anaplastic Large Cell Lymphoma. Glandular atrophy and encapsulation can develop potentially delaying cancer diagnosis.^[3]^

Surgically, migration of the gel almost always prevents complete removal and often requires extensive reconstructive techniques. Considering PAAG may have potential toxicity and radiological interpretation is significantly compromised, careful assessment is required to understand how best to manage this group of patients often presenting with multiple complications; the long term implications are yet unknown.

[1] Wang Z, Li S, Wang L, Zhang S, Jiang Y, Chen J, Luo D. Polyacrylamide hydrogel injection for breast augmentation: another injectable failure. Medical science monitor: international medical journal of experimental and clinical research. 2012;18(6):CR399.

[2] Luo SK, Chen GP, Sun ZS, Cheng NX. Our strategy in complication management of augmentation mammaplasty with polyacrylamide hydrogel injection in 235 patients. Journal of Plastic, Reconstructive & Aesthetic Surgery. 2011 Jun 1;64(6):731-7.

[3] Winter J, Shiga S, Islur A. The Complications of Polyacrylamide Hydrogel Augmentation Mammoplasty: A Case Report and Review of the Literature. Plastic Surgery Case Studies. 2017 Mar 10;3:2513826X17693821.

## P53 A planned feasibility and pilot study to compare two mammographic imaging protocols in women with breast implants

### Alex Coltart

#### University Hospital Crosshouse, Crosshouse, United Kingdom

**Background:** Scant literature on optimal mammographic imaging for women with breast implants exists beyond that published by Eklund et al in 1988^[1]^, so there is little evidence on which to base best practice. This study aims to establish the feasibility of a larger study to compare two “Eklund-based” mammography protocols for screening women with breast implants, specifically:

1. whether acceptable levels of intra-rater reliability (single consultant radiographer rater) on the outcome measures can be achieved and

2. participant recruitment rate. Pilot data will be collected to enable a sample-size calculation for a future definitive study comparing the amount of breast tissue demonstrated and the mean glandular dose received by the two mammographic protocols.

**Methods:** The two protocols to be compared are

1. Standard series - medio-lateral oblique (MLO) and cranio-caudal (CC) implant-in-field, plus implant-displaced versions of both projections (current standard practice in Scotland - 8 views total);

2. Alternative series – standard MLO implant-in-field and MLO implant-displaced, plus implant-displaced medially- and laterally-extended CC (i.e. no standard CC views); also 8 views total.

Following NHS Research Ethics Service and management approvals, a repeated measures design will be used, i.e. participants as their own controls. With written informed consent, 20 participants will have the Scottish standard 8-view mammographic series, plus two extended implant-displaced CC views to one breast. The standard versus alternative series of views for the 20 single breasts will be compared for: area of displaced tissue demonstrated, depth of tissue visualised between the nipple and the chest wall, radiation dose.

[1] Eklund, G.W., Busby, R.C. et.al (1988). Improved Imaging of the Augmented Breast. *American Journal of Roentgenology*, 151, pp.469-473

## P54 Retrospective study of the visibility of subtle abnormalities with the Eklund view in mammography in women with breast implants

### Anuma Shrestha, Francesca Moakes

#### **Correspondence:** Anuma Shrestha

##### University College London Hospital NHS Foundation Trust, London, United Kingdom

**Purpose:** Insertion of breast implants (BI) is a commonly performed procedure either for cosmetics reasons or for reconstruction following mastectomy [1]. BI reduces the sensitivity of the mammogram to breast tissue being obscured by the implant [2]. The Eklund Technique (ET) performed in addition to routine mammograms uses a pushed back technique to provide good compression of the anterior breast tissue to improve visibility of subtle breast abnormalities [3]. We carried out a retrospective review of 79 patients to evaluate the visibility of lesions in ET compared with standard 2 view mammograms in symptomatic settings between May 2018 to Jan 2020.

**Methods:** All images were reviewed on a mammogram approved high resolution 12-megapixel Barco monitor. The routine mammogram images were compared with the ET to assess visualisation of subtle lesions: microcalcification, distortion, mass and asymmetric densities. The finding was compared with final double report of the consultant radiologist/radiographer including further views/tomosynthesis/ultrasound and histopathology outcomes. Microsoft Excel (2010) was used for statistical analysis.

**Results:** 14 cases showed extra details in ET with better visibility of lesions out of 79 when compared with ordinary 2 views (7 microcalcification, 2 distortion and 5 mass). Total 5 cases underwent biopsy (3 microcalcification were benign, 1 distortion and 1 mass were malignant); 9 cases had benign mammographic features.

**Conclusion:** ET has shown improved visibility of subtle lesions predominantly microcalcifications. ET with breast implants substantially improved image quality and also helped to eliminate diagnostic dilemma.

[1] Kam K, Lee E, Pairawan S, Anderson K, Cora C, Bae W, Senthil M, Solomon N, Lum S. The Effect of Breast Implants on Mammogram Outcomes. 2015 October; The American Surgeon. 81 (10), pp. 1053-1056(4).

[2] Miglioretti D L, Rutter CM, Geller BM, Cutter G, Barlow WE, Rosenberg R, Weaver DL, Taplin SH, Ballard-Barbash R, Carney PA, Yankaskas BC, Kerlikowske K. 2004 Jan. Effect of Breast Augmentation on the Accuracy of Mammography and Cancer Characteristics. *JAMA.*291(4):442-450.

[3] Venkataraman S, Hines N and Slanetz PJ. Challenges in Mammography: Part 2, Multimodality Review of Breast Augmentation—Imaging Findings and Complications. American Journal of Roentgenology.AJR Am J Roentgenol.2011 Dec; 197(6): W1031-45.

## P55 How can a breast screening service support women who have a learning disability to access breast screening?

### Sarah Lea, Claire Bailey, Susan Harley

#### **Correspondence:** Sarah Lea

##### South West London Breast Screening Service, St George’s Hospital NHS Trust, London, United Kingdom

**Background:** Evidence shows that women with a learning disability are more prone to developing cancer than the general population. ^[2]^ A lack of reasonable adjustments can be a barrier to accessing healthcare settings. ^[1].^ It is important that a breast screening service makes adaptations to assist women with a learning disability access screening.

**Method:** A review of a Breast Screening service practice and the adaptations that have been made within the service to facilitate women with a learning disability access screening was carried out. Adaptations included developing a database and improving the process of recording client activity and asking for assistance from general practitioners in identifying women with a learning disability. Changes to the actual screening process were also made. The screening uptake figures for women with a learning disability compared uptake in 2016-17 with the uptake in 2018-19 to see if there had been any improvement.

**Results:** The breast screening uptake for women with a learning disability improved in 2018-19 compared with 2016-17 which indicated that the interventions had been beneficial. The new interventions which were introduced became part of a set of work instructions incorporated into the clinical practice of the unit.

**Conclusion:** In reviewing the adaptations that had been made to facilitate screening for women with learning disabilities we identified measures that appeared to make a difference according to the uptake figures.

[1] Ali, A., Scior, K., Ratti, V., Strydom, A., King, M., & Hassiotis, A. (2013). Discrimination and other barriers to accessing health care: perspectives of patients with mild and moderate intellectual disability and their carers. *PloS one*, 8(8).

[2] Heslop, P., Blair, P., Fleming, P., Hoghton, M., Marriott, A., & Russ, L. (2013). Confidential Inquiry into premature deaths of people with learning disabilities (CIPOLD). *Bristol: Norah Fry Research Centre*.

## P57 Effective implementation of an advanced clinical practitioner role in breast imaging

### Linda Deane, Sue Williams, Lucy Cielecki, Sian Burley

#### **Correspondence:** Linda Deane

##### Shrewsbury and Telford Hospital NHS Trust, Wellington, United Kingdom

**Background:** Due to the immense pressure to provide capacity for women with breast symptoms, to be seen within two weeks, a new innovative role has been created to provide increased capacity.

**Introduction:** The breast services see many women with conditions that are benign and easily identified upon ultrasound. The majority of these conditions occur in women under the age of 40years.

The role of an advanced clinical practitioner was created to answer a service need. This role requires a highly specialised cohort of skills combining breast image interpretation, breast ultrasound and breast biopsying alongside a range of clinical competences enabling autonomous practice within clear governance.

**Methods:** A new clinic was created for under 40 aged women only requiring only a breast clinical specialist and an advanced clinical practitioner, using ultrasound for assessment. Unexpected findings- suspicious upon ultrasound- would be redirected to the next consultant led clinic for full imaging assessment and biopsy.

**Results:** Increased capacity was achieved, without increased costs.  Anxiety levels were reduced due to these patients seen within these clinics and more specialist skills could be directed to more complex cases in the traditional cancer clinics.

**Conclusion:** The use of this specialist role has proven to be innovative and specialised in answering capacity issues within the workforce. The ACP role is utilised as a support to all clinics working alongside consultant radiographers as well as in an autonomous role, thereby freeing up the consultants for cases requiring specialist skills. The stability of the breast service has been ensured.

## P58 Abreast of the times

### Katherine Barton, Victoria Bremner

#### **Correspondence:** Victoria Bremner

##### Portsmouth Hospitals University NHS Trust, Portsmouth, United Kingdom

**The Problem**: The shortage of breast imaging staff is both a national and local issue; in 2016 a Public Health England survey [1] reported a national vacancy rate for breast imaging practitioners of 15%.

**A Solution?** In an attempt to overcome this issue locally, our breast unit has implemented an ongoing training programme to train 9 mammographers in the 3½ year period to September 2021, utilising both the post graduate route for qualified radiographers and the new Mammography Associate apprenticeship. In addition, two experienced mammographers have been supported to qualify as advanced practitioners in mammography, providing additional support for our radiologists. This has required a strategic and collaborative approach, involving all staff groups. In order to ensure that all trainees achieve competency and receive a quality training experience the following strategies have been employed:

- Full staff engagement
- Dedicated mentors and practical trainers
- Locum staff for backfill
- Close collaboration with the breast screening administrative team to provide dedicated training lists
- Individualised timetables for trainees to maximise training opportunities and ensure an equitable training experience for all
- Timetabled study leave - to minimise students feeling overwhelmed

**Where are we now?** At the time of writing 5 trainees have successfully completed their training and a further 2 radiographers and 2 mammography assistant practitioners are due to complete their studies within the next twelve months. This will mean that the unit has a full compliment of mammographic staff to support our expanding service and will allow more experienced staff to develop into advanced practitioner roles.

[1] Public Health England. NHS Breast Screening Programme National radiographic workforce survey. 2016. Available from: https://www5.shocklogic.com/scripts/jmevent/Abstract_2.asp?Client_Id='PP'&Project_Id='SYMP20AB'&Crypt=UGVyc29uX0lkPTQ5ODYyMjYmRm9ybV9JZD0mRm9ybV9OdW1iZXI9MiZMYW5ndWFnZV9Db2RlPSZBPQ== [Accessed 07 October 2020].

## P59 A case study focusing on invasive ductal carcinoma occurring in a male patient

### Rachel Tilburn^1^, Claire Mercer^2^

#### Rachel Tilburn

##### ^1^University Hospitals of Morecambe Bay, Kendal, United Kingdom; ^2^University of Salford, Salford, United Kingdom

**Background:** This is a presentation of a case study focusing on a male patient with invasive ductal carcinoma. This work focuses on a real-life scenario, showing delayed presentation of symptoms, guidelines, surgical options and the male patient experience, as well as demonstrating how the clinical pathway has been adapted within a national pandemic.

**Methods:** A male in his eighties was referred from their General Practitioner (GP) within NICE guidelines ^[1]^. Male breast cancer is rare, currently accounting for less than 1% of all breast cancers, with the lifetime risk of diagnosis around 1 in 833 ^[2]^. Due to lack of mammographic screening for this cohort of patients, public awareness is low and any cancers diagnosed often present at an advanced stage ^[3]^.

**Outcome**: Following triple assessment, Grade 2 Invasive Ductal Carcinoma (IDC), B5b was diagnosed. Originally the patient was due to undergo a nuclear medicine scan and mastectomy, however due to the Coronavirus outbreak; this was cancelled. To overcome the problem of surgery being delayed, it was decided within an online MDT that the patient should be started on early hormone therapy, this began swiftly.

**Conclusions**: By highlighting the need for increased awareness and helping combat the stigmatism in relation to male breast imaging, it should in turn support early diagnosis and hopefully encourage males to have open discussions around their diagnosis, reducing the embarrassment which currently surrounds this disease. This work also encourages the discussion around gaps in current research, emphasising what new research needs to be undertaken and what impact this could have.

[1] National Institute for Health and Care Excellence. Suspected Cancer Recognition and Referral [Internet]. London: National Institute for Health Care Excellence; 2015 [updated 2017 July 26; cited 2020 June 17]. Available from: https://www.nice.org.uk/guidance/ng12/resources/suspected-cancer-recognition-and-referral-pdf-1837268071621

[2] Breast Cancer. Male Breast Cancer [Internet]. Pennsylvania: Breast Cancer; 2020 [updated 2020 Jan 27; cited 2020 June 17]. Available from: https://www.breastcancer.org/symptoms/types/male_bc

[3] Incorvaia L, Castiglia M, Ottini L, Gori S, Russo A, Bazan V. Plastic and Cosmetic Surgery of the Male Breast [Internet]. Switzerland: Springer; 2020 [cited 2020 June 18]. 170 p. Available from: https://link.springer.com/book/10.1007%2F978-3-030-25502-2#toc doi: 10.1007/978-3-030-25502-2

## P60 The development and implementation of the mammography associate apprenticeship

### Lyndsay Kinnear

#### National Breast Imaging Academy, Manchester, United Kingdom

Shortages across the breast imaging workforce have had an impact on providing timely and efficient screening services to women.

This poster presents the development and implementation of the Mammography Associate Level 4 Apprenticeship. An overview of the trailblazer group which brought together mammography professionals from NHS trusts across the country, academic institutions and the Society and College of Radiographers, gaining Institute for Apprenticeships approval and the logistics of delivering the training is summarised. In addition to this the poster will present a case study of a student who has completed the Mammography Associate Apprenticeship.

In anticipation of a potential influx of Mammography Associates following the report from Public Health England which provides information on allowing experienced assistant practitioners (APs) to work on mobile vans or remote static sites ^[1]^, this poster educates service managers and alike to consider this role within their teams and provides a broader understanding of the development of the apprenticeship, funding training via the apprenticeship levy and the success of this training programme. Along with further insight into the ease of recruitment of this staff group and standardising training.

[1] https://www.gov.uk/government/publications/breast-screening-assistant-practitioner-pilot-report

## P62 The credential in breast disease management: A trainee breast clinician's experience of a new training pathway

### Mayada Haydar, Caroline Parkin

#### **Correspondence:** Mayada Haydar

##### Manchester University NHS Foundation Trust, Manchester, United Kingdom

In response to the current workforce crisis that is affecting breast imaging services, a new credential in breast disease management was developed by the Association of Breast Clinicians (ABC), Royal College of Radiologists (RCR), National Breast Imaging Academy (NBIA) and Health Education England (HEE) to train breast clinicians ^[1]^. This poster presents the experience of a trainee breast clinician enrolled in the first cohort of the national pilot after starting their training in Manchester in August 2019. An overview of the structure of training and assessment, the educational and support resources used and personal reflections on the highlights and challenges experienced is summarised. In anticipation of expansion of the training programme, this poster educates people considering this career pathway for themselves, staff at breast units considering joining the training scheme and the broader breast community in understanding the scope and success of this innovation in training.

[1]. Royal College of Radiologists website [Internet] cited 2020 Oct 14th. Available from: https://www.rcr.ac.uk/clinical-radiology/careers-recruitment/breast-clinicians-credential-breast-disease-management

## P63 Does arbitration work?

### Susan Williams, Linda Deane, Sian Burley, Lucy Cielecki, Umit Aksoy, Marie Metelko

#### **Correspondence:** Susan Williams

##### Shreswbury and Telford Hospital NHS Trust, Shrewsbury, United Kingdom

**Introduction:** To improve cancer detection rates, personal performance and as part of our routine service improvement programme, an audit was undertaken of discordant cases returned directly to routine recall between 1/4/15 and 31/3/17 inclusive. These were reviewed against the results of the subsequent screening round to determine if the correct judgement had been made at the previous screening round or if there were any opportunities to learn from misinterpretation.

**Method:** All cases arbitrated and directly returned to routine screening between 2015/16 and 2016/17 were identified and cross-referenced with the results for the subsequent screening episode. All screen detected cancers previously arbitrated on the same side were reviewed by the same routine method and criteria as all interval cancers within our unit and each was given an 'interval' category. All of the screen detected cancers previously arbitrated on the same side were included in the annual interval cancer review session to discuss learn opportunities and improved outcomes.

**Results:** There were 829 cases arbitrated and returned to routine screening at the original screening episode 2015/16 or 2016/17. 11 cases were diagnosed with a same side screen detected cancer at the subsequent screening round and 2 cases presented as a same side interval cancer. Neither interval cancers detected at the case review. 1 of the 11 same side screen detected cancers classified as minimal signs.

**Conclusion:** In our unit arbitration cases returned to routine recall is the correct decision in the vast majority

## P64 Standout and be outstanding mammographer redeployment during the Covid-19 crisis

### Adele Fairburn, Tania Brosnan

#### **Correspondence:** Adele Fairburn

##### York Teaching Hospital Foundation Trust, York, United Kingdom

**Objective:** The purpose of this research is to investigate the impact of redeployment and the mental health issues affecting redeployed Mammographers during the Covid-19 Crisis.

**Method:** A focused, survey-based, national study collating mental health information and demographic data from 90 UK redeployed Mammographers.

**Results:**

- 50:50 voluntary redeployment.
- 8+ weeks average length of redeployment.
- 22.09% felt a moral duty to redeploy.
- 17.94% developed feelings of anxiety.
- 67% pleased to return back to Mammography.
- 26.97% reconsidered their role.
- 63.33% had family support to help cope with redeployment.
- 73.33% had no access to mental health support.
- 74.44% stated redeployment would have a lasting impact.
- 67.78% felt the experience was positive, 15.45% negative.

**Conclusions:** Health care workers on the front line are at risk of developing psychological distress and other mental health symptoms.^[1]^ Our research demonstrated that the mental health of Mammographers was affected by redeployment. Whether their experiences were positive or negative was directly proportional to the level of support offered to them by their colleagues and managers. The majority of redeployed staff were welcomed and supported by their colleagues which contributed towards a positive learning experience and post-traumatic growth.^[2]^

Our research found that the majority of respondents had no mental health support post redeployment. Trusts need to act quickly to ensure the health care professionals they rely on during such times of crisis are given the post traumatic support they both need and deserve, to help them recover from the events they have experienced.

[1]. Jianbo Lai, Simeng Ma, Ying Wang, etal. Factors Associated With Mental Health Outcomes Among Health Care Workers Exposed to Coronavirus Disease 2019. JAMA Network, 2020;(3):e203976.doi:10.1001/jamanetworkopen.2020.3976

[2]. Greenbeg N., Docherty M., Gnanapragsam S., Wessley S., Managing mental health challenges faced by healthcare workers during Covid-19 pandemic. BMJ, March 2020 ;368:m1211

[4]. Rimmer A, How can I cope with redeployment? *BMJ* 2020;368:m1228 doi 10.1136/bmj.m1228

